# Aberration-corrected hybrid metalens for longwave infrared thermal imaging

**DOI:** 10.1515/nanoph-2023-0918

**Published:** 2024-06-03

**Authors:** Tie Hu, Liqing Wen, Haowei Li, Shengqi Wang, Rui Xia, Zihan Mei, Zhenyu Yang, Ming Zhao

**Affiliations:** School of Optical and Electronic Information, 12443 Huazhong University of Science and Technology, Wuhan 430074, China; School of Engineering and Applied Science, 5755 Yale University, New Haven, CT 06520, USA

**Keywords:** hybrid metalens, aberration-corrected, thermal imaging, longwave infrared

## Abstract

Wide-angle metalenses in the longwave infrared have shown great advantages over the traditional refractive doublets or triplets, due to light weight, CMOS compatibility, and low cost. However, previous endeavors have been plagued by challenges including a narrow waveband, large F-number, distortion, and spherical aberration. To address these problems, this study introduces two dispersive metasurfaces, placed near the front focal plane and upon the rear plane of a plano-convex lens, to correct optical aberrations. Utilizing this methodology, we propose and experimentally demonstrate an aberration-corrected hybrid metalens for thermal imaging in the 8–12 μm waveband, featuring an FOV of 24°, F-number of 1.2, and diameter of 12.2 mm. The developed hybrid metalens rigorously evaluated, exhibits Modulation Transfer Function (MTF) values exceeding 0.2 at 20 Lp/mm across the full FOV, and features an average transmission of 48.7 %, a relative focusing efficiencies of up to 42.1 %, polarization insensitivity and broadband imaging capacity. These results emphasize the potential applications of our system in diverse fields, such as camera lenses, autonomous driving, healthcare, and environmental monitoring.

## 1 Introduction

Longwave infrared (8–12 μm) thermal imaging is crucial in various domains, including healthcare, autonomous driving, smart home systems, and facility maintenance [1]. Advancements in these areas increasingly demand thermal imaging lenses with high performance, light weight, compactness, and low cost [2]. The conventional method, comprising multiple refractive lenses with different materials and complex aspherical surfaces, corrects optical aberrations but leads to bulkiness, high cost, and low yield [3], [4]. In contrast, metalenses, featuring diffraction-limited focusing and offering ultra-compactness and CMOS compatibility, provide a promising solution for high-performance thermal imaging lens [5], [6], [7]. However, they face challenges with monochromatic aberrations like coma, astigmatism, distortion, and spherical aberration, as well as chromatism [8].

Over the past decade, numerous studies have been conducted to achieve achromatism of single metalens, such as dispersion engineering [9], [10], [11], annular interference [12], [13], spatial multiplexing [14], [15], computational optimization [16], [17], and multi-layer metalens [18]. However, these methods involve trade-offs among numerical aperture, diameter, efficiency, and working bandwidth due to the insufficient phase dispersion from the subwavelength meta-atoms [19], [20]. To overcome this limitation, metasurface-refractive hybrid metalenses, combining refractive lenses for primary optical power with dispersion-engineered metasurfaces to counteract the positive dispersion of refractive lens, have been introduced [21], [22], [23]. For instance, Hu et al. developed a centimeter-scale achromatic hybrid metalens with an F number of 2.64 in the visible, whereas there is still monochromatic aberration introduced by the non-paraxial effect for large field-of-view (FOV) [23]. To eliminate monochromatic aberrations in metalenses, three approaches – multi-layer topological metalenses, single-layer quadratic metalenses, and double-sided metalenses – have shown promise in achieving a large FOV [24]. By optimizing the angular dispersion of multilayered metasurface topologically, Lin et al. proposed a 22*λ* sized metalens capable of diffraction-limited focusing across a 40 × 40° FOV at a specific wavelength [25]. Alternatively, single-layer quadratic metalenses utilize intrinsic symmetry transformation to suppress coma aberration, but still suffer from spherical aberration, distortion, and chromatism [26], [27], [28]. Inspired by the design of the traditional landscape lens, double-sided metalenses are proposed to compensate for off-axis aberrations with the sacrifice of its effective aperture [29], [30], [31]. For example, Amir Arbabi et al. demonstrated a miniature camera at the wavelength of 915 nm with a diameter of 0.8 mm and an FOV of 60° × 60° [29]. Some similar approaches, such as multi-level diffractive lenses [32], [33] and compound eye metalens arrays [34], [35], have also been explored. To date, despite various proposed methods, the realization of aberration-corrected metalens with centimeter-scale diameter, and a broadband working bandwidth is well in need.

In this paper, we respectively place two metasurfaces near the front focal plane and upon the rear plane of a plano-convex lens and experimentally demonstrate an aberration-corrected polarization-insensitive hybrid metalens, with an F number of 1.2, a diameter of 12.2 mm, and an FOV of 24 × 24° for the longwave infrared wavelength from 8 μm to 12 μm. Unlike previous works, our hybrid metalens uses two metasurfaces to correct the chromatism and monochromatic aberrations of a single plano-convex lens, while keeping the same diameter of the three optical elements without sacrificing the effective aperture of the system. The reported hybrid metalens is carefully evaluated and exhibits an average transmission of 48.7 %, a relative focusing efficiencies of up to 42.1 % more than double that of the plano-convex lens, an MTF larger than 0.2 at 20 Lp/mm across the full FOV, and better imaging capabilities compared with a plano-convex lens.

## 2 Design and fabrication

### 2.1 Design and optimization of the hybrid metalens

**Figure 1:**
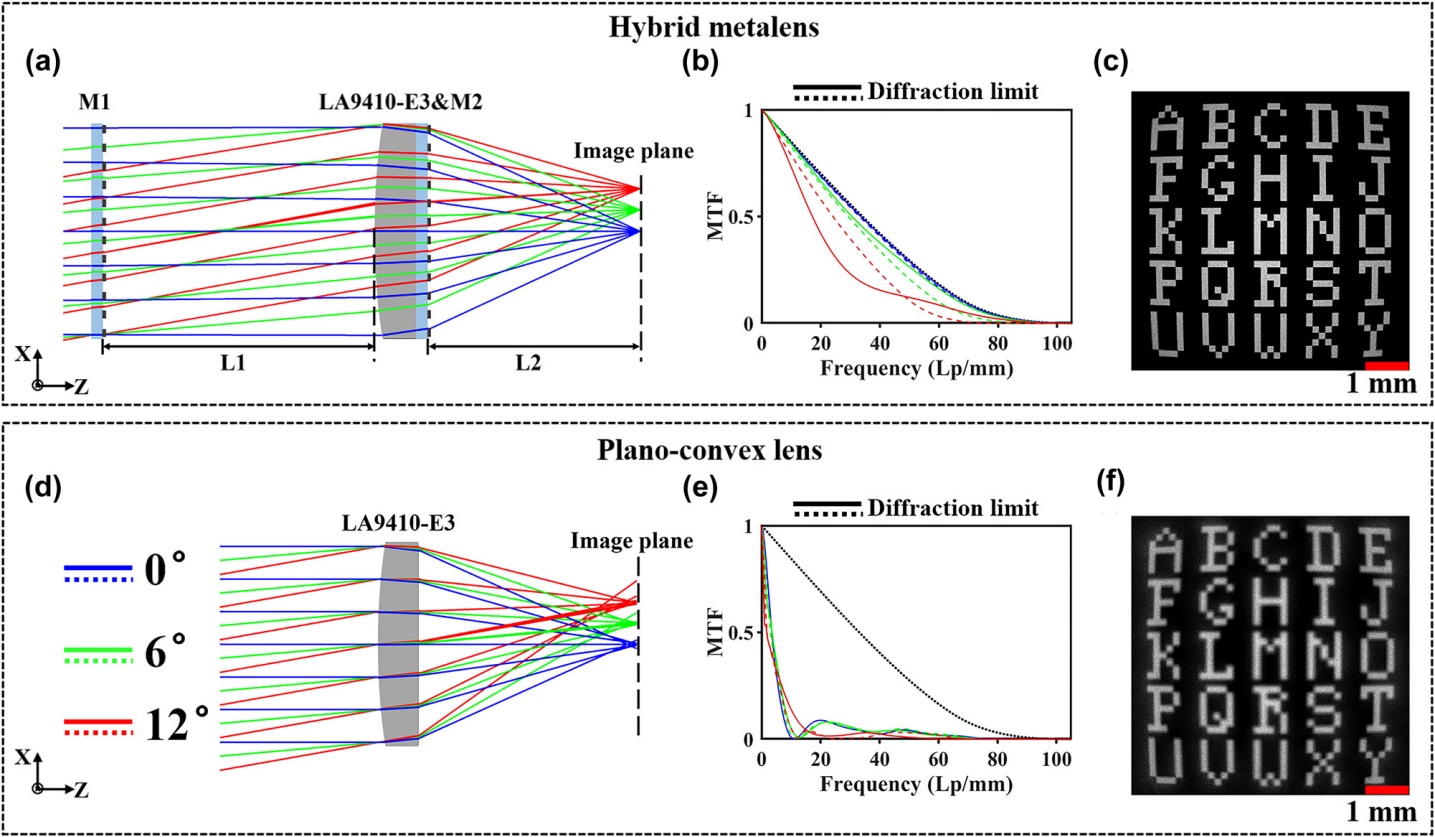
Design of the hybrid metalens and performance comparison with plano-convex refractive lens (Thorlabs, LA9410-E3). (a) Scheme of the hybrid metalens, composed of two aberration-corrected metasurfaces and a plano-convex refractive lens LA9410-E3. L1 and L2 respectively denote the distances from metasurface M1 to the front of LA9410-E3 and from M2 to the focal plane. (d) Geometric focusing effect of LA9410-E3. (b), (e) MTF curves vice incidence angles: (b) Hybrid metalens. (e) LA9410-E3. Blue, green, red, and black lines represent the results of incident angle 0°, 6°, 12°, and the diffraction limit, respectively. Imaging characterizations for (c) hybrid metalens. (f) LA9410-E3. Scale bar: 1 mm.

As depicted in Figure 1(a), the metalens comprises a germanium (Ge) plano-convex refractive lens (Thorlabs, LA9410-E3) paired with two Ge metasurfaces, each sharing the same diameter as the refractive lens. Inspired by the Schmidt corrector plate design, metasurface M1 located at distance L1 from the front of the LA9410-E3, tackles most off-axis aberrations, while M2 placed upon the flat surface of LA9410-E3, mitigates residual monochromatic aberrations. Additionally, the negative dispersion of both metasurfaces neutralizes the positive one of the LA9410-E3, thereby effectively eliminating chromatic aberration. Phase profiles for both metasurfaces, along with distances L1 and L2, are optimized via the ray tracing method. Here, L2 is the distance from metasurface M2 to the focal plane, optimized to 16.4 mm for L1 and 12.8 mm for L2. Further details on the optimization methods can be found in Supplementary Material S1. To demonstrate the effectiveness of our design, the simulated and experimental optical performance of the hybrid metalens is compared against that of LA9410-E3.

The hybrid metalens and LA9410-E3 are numerically analyzed with Zemax OpticStudio (Ansys, Inc.) for various optical characteristics. Standard spot diagrams in Supplementary Material Figure A2 and RMS radii from Supplementary Material Table A2 illustrate the geometric focusing effects. In Supplementary Material Figure A2(a), the spot diagrams of the hybrid metalens show concentrated patterns within the Airy ring, contrasting with the widely scattered spots of LA9410-E3 in A2(b). This is reflected in the fact that the RMS radii of the hybrid metalens are significantly smaller, while the corresponding values for LA9410-E3 are considerably larger than the Airy radius. Figure 1(b) and (e) display broadband MTF curves for the hybrid metalens and LA9410-E3, respectively. The MTF of LA9410-E3 drops sharply to 0.1 at 9 Lp/mm, while the hybrid metalens decreases gradually to 0.1 at 52 Lp/mm across the full FOV, indicating its superior resolution and aberration correction. As per convention, the broadband encircled energy listed in Supplementary Material Table A3 quantitatively assesses lens efficiency. At the encircled radius of 12 μm, the encircled energy of the hybrid metalens reaches up to 81 %, nearly tenfold that of LA9410-E3 across the full FOV, revealing its superior efficiency. Additionally, Figure 1(c) and (f) display alphabet letter chart tests over a 24° FOV, using broadband light. Figure 1(c) shows the hybrid metalens producing a sharp, minimally aberrated image, unlike the severely blurred image from LA9410-E3 in Figure 1(f). Overall, the hybrid metalens significantly outperforms the plano-convex lens in aspects like RMS radius, encircled energy, resolution, and geometric imaging.

**Figure 2:**
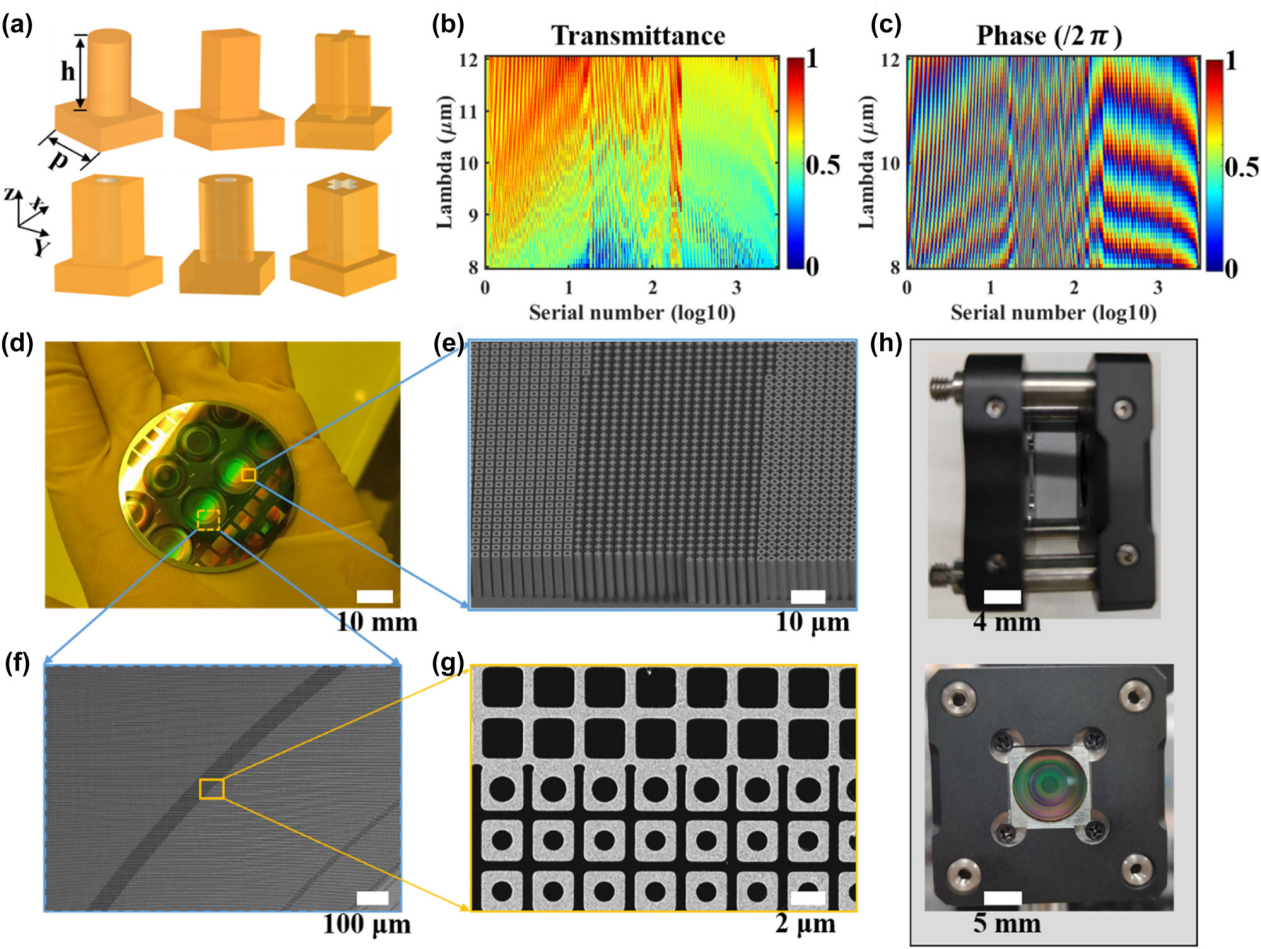
Metasurface design and manufacturing. (a) Illustration of the six types of meta-atoms. (b), (c) Simulated transmittance and phase shift of the meta-atom library under the *x* linearly polarized light. (d) Optical image of the fabricated germanium metasurface wafer. Scale bar: 10 mm. (e)–(g) Scanning electron micrographs (SEMs) of (e) the tilted view for (d), (f) the top-view for the metasurface M2 (yellow dashed box in (d)), (g) magnified view in (f). Scale bars are respectively 10 μm, 100 μm, and 2 μm for (e)–(g). (h) Mechanical Assembly of the hybrid metalens. Scale bar: 5 mm.

To achieve the desired phase profiles, the metasurfaces consist of different Ge meta-atoms shown in Figure 2(a), composed of Ge nanopillars with fixed heights of 16 μm and periods of 3 μm upon the Ge basement. More details of meta-atom design can be seen in Supplementary Material S2. The four-fold symmetry of all meta-atoms ensures polarization insensitivity. By the finite time domain difference (FDTD) method, the simulated transmittance and phase shift of various meta-atoms are collected in Figure 2(b) and (c), respectively. A global optimization algorithm is developed to select the proper meta-atoms for each position on the metasurfaces, aiming to minimize the average phase error [23]. Supplementary Material Figure A4 illustrates the achieved phase and transmittance distributions of the two metasurfaces. The maximum RMS wave aberration function (WAF) for both metasurfaces remains under 0.057 wave across the entire working waveband, meeting the Maréchal criterion $\left(\text{WAF}\leq \frac{1}{14}\right)$ . These results verify the successful implementation of the designed metasurfaces.

### 2.2 Device fabrication

We experimentally developed the hybrid metalens using a 2-inch Ge wafer, defining the meta-atom patterns by ultraviolet lithography and Bosch dry etching (more details can be seen in Supplementary Material S4). Figure 2(d)–(g) show the optical image and scanning electron micrographs (SEMs) of the patterned wafer in various magnifications and views. These figures highlight the high precision of the fabrication process, evidenced by a large sidewall angle of 89.8°, an aspect ratio of 38.6:1, and a critical dimension as small as 0.4 μm. Prior to scoring, a layer of photoresist film is spin-coated on the patterned wafer to protect the fabricated metasurfaces. Following scoring and photoresist removal, two selected metasurfaces are assembled with LA9410-E3 to form the designated hybrid metalens (Figure 2(h)). As seen in Supplementary Material S5, the experimental feasibility of the hybrid metalens is demonstrated by the analysis of displacement tolerance and achievable mechanical accuracy.

## 3 Results and discussion

### 3.1 Focusing with the hybrid metalens

**Figure 3:**
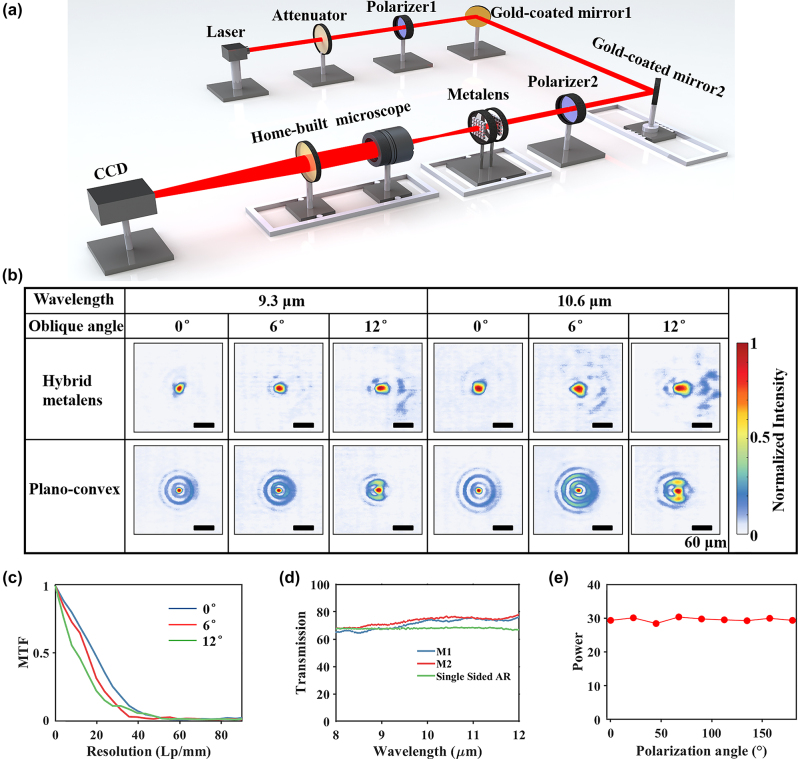
Optical characterization of the hybrid metalens. (a) Scheme of the optical setup. (b) Normalized intensity distributions versus incident angle and light source wavelength. Scale bar: 60 μm. (c) Polychromatic MTF curves under different incident angles for the hybrid metalens. (d) Measured transmittances. Blue, red, and green lines denote the results of the metasurfaces M1, M2, and Ge substrate with single-sided anti-reflective film, respectively. (e) Power with the incident polarization angle.

For focusing performance evaluation, collimated and expanded beams with incident angles ranging from −12° to 12° are used to illuminate the test lenses at wavelengths of 9.3 μm and 10.6 μm (see Figure 3(a)). See Supplementary S7 for experimental details. Figure 3(b) shows the point spread functions (PSFs) of the two lenses, indicating variations at different incidences (0°, 6°, and 12°) and wavelengths (9.3 μm and 10.6 μm). As the incident angle increases, the hybrid metalens exhibits expanding focal spots and deteriorating sidelobes. Specifically, the full width at half maximum (FWHM) grows from 18.06 μm to 36.49 μm as the incident angle increases from 0° to 12°. Minor lateral alignment errors between the two metasurfaces in the hybrid metalens may cause slightly asymmetric focal spots. Compared with the focal spots of LA9410-E3, the hybrid metalens shows lower sidelobes at all angles and wavelengths, indicating better aberration suppression, especially spherical aberration. Figure 3(c) presents the polychromatic MTF curve for the hybrid metalens at various incident angles. MTF values decrease with increasing incident angles for each spatial frequency, consistent with previously discussed simulations. The hybrid metalens also maintains MTF values above 0.2 at 20 Lp/mm across the entire FOV. Slightly lower MTF values observed here are attributed to imperfect manufacturing and alignment errors. Specifically, Supplementary Material Figure A10 indicates that all MTF values decrease by more than half as transverse alignment error ranges from 0 to 0.5 mm across the entire FOV. As shown in Supplementary Material Figure A11, large etching depth variations stem from the inherent etching depth differences between nanopillars and nanoholes, and the microloading effect linked to their varying aspect ratios. Given the fabrication errors, we simulate the optical responses of the hybrid metalens along a certain diameter, revealing significant fluctuations and deviations in the phase profiles of both metasurfaces (see Supplementary Material Figure A13). Additionally, in Supplementary Material Figure A14, the hybrid metalens with the microloading effect has significantly inferior resolution compared to the one without it. For instance, the MTF of the hybrid metalens with the microloading effect sharply drops to 0.1 at 10 Lp/mm, while without this effect, it gradually decreases to 0.1 at 52 Lp/mm across the full FOV.

Lens efficiency is determined by its transmittance and relative focusing efficiency. Here, the relative focusing efficiency is defined as the power ratio within a circular area, three times the diameter of the Airy spot radius, to the total power at the focal plane. Figure 3(d) displays the measured transmission spectra of the two metasurfaces and the Ge wafer, averaging transmittances of 71 %, 73 %, and 68 %, respectively. Considering LA9410-E3 has an average transmittance of around 94 %, the hybrid metalens achieves an average transmittance of roughly 48.7 %. Notably, the substrate of the two metasurfaces and one side of Ge wafer are coated with a longwave infrared broadband anti-reflective film. In the hybrid metalens, the average relative focusing efficiency surpasses LA9410-E3 by over double: 42.1 % at 9.3 μm and 34.6 % at 10.6 μm (see Table 1). Assuming absolute focusing efficiency equals the product of transmittance and relative focusing efficiency, it is 20.5 % at 9.3 μm and 16.9 % at 10.6 μm. To improve efficiency, the hybrid metalens will employ specially designed meta-atoms and enhance the alignment accuracy, as demonstrated by the simulated encircled power results in Supplementary Material S5.

**Table 1:** Comparison of the relative focusing efficiency.

Angle					
Wavelength		0°	6°	12°	Average
9.3 μm	Hybrid metalens	57.4 %	43.4 %	25.4 %	42.1 %
	Plano-convex	16.8 %	16.2 %	14.2 %	15.7 %
10.6 μm	Hybrid metalens	36.6 %	31.4 %	35.7 %	34.6 %
	Plano-convex	18.0 %	14.0 %	11.4 %	14.5 %

To characterize the polarization insensitivity, we employ a precise electric rotary stage to align the metalens orientations with the *X*-axis forward direction. In Figure 3(f), powers at various polarization angles show a shift of ±4 % from the average of 29.34, indicating the polarization-insensitivity of the hybrid metalens. Here, power is defined as the total light intensity within an 18 × 18 pixel window at the focus center, with power shift being the percentage of the difference between the measured power and average power to the average power. As listed in Supplementary Material Table A6, the focal length shift of about 30 μm is negligible relative to the effective focal length of 14.8 mm in the 9.3–10.6 μm waveband, underscoring the achromatism of the hybrid metalens.

### 3.2 Imaging performance

**Figure 4:**
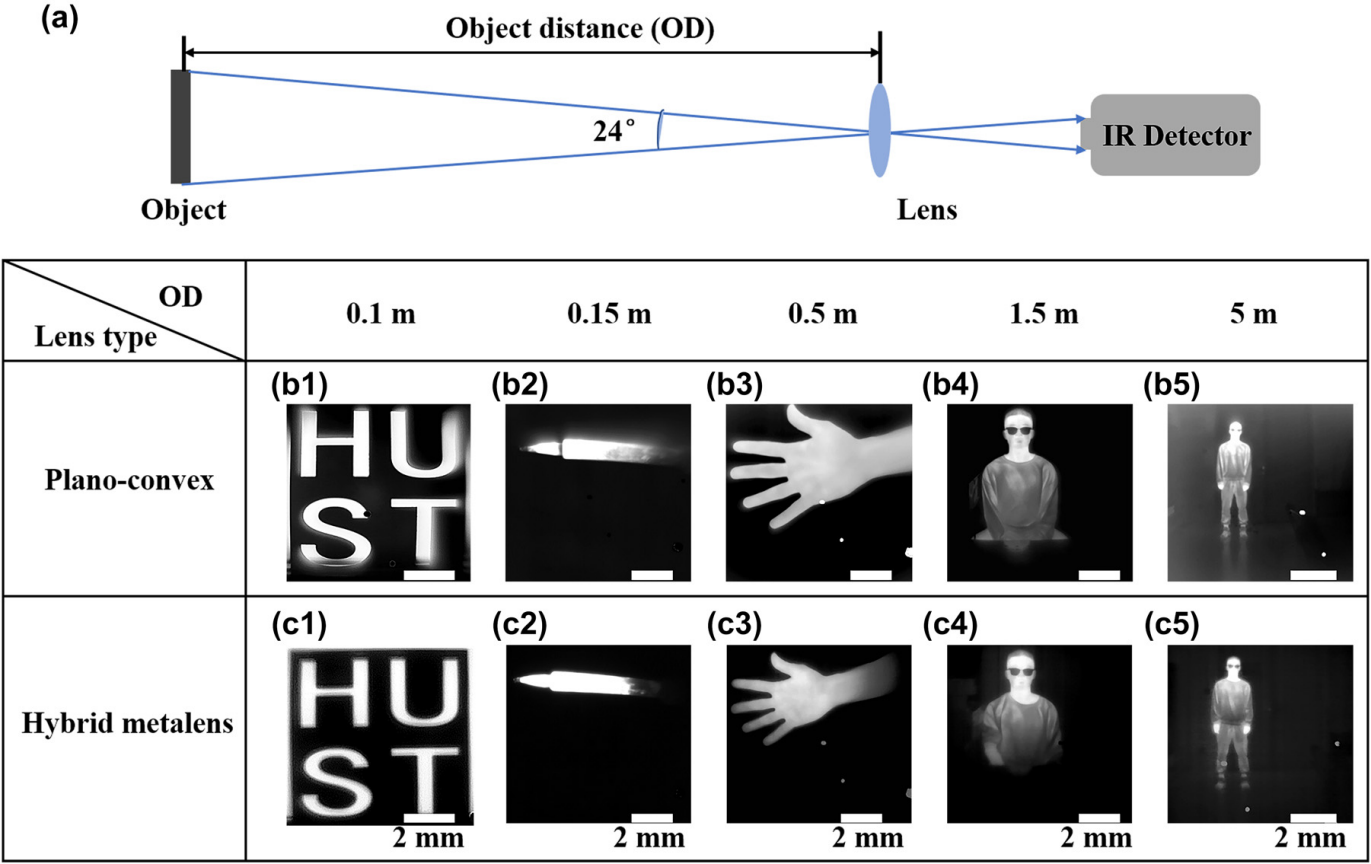
Imaging characterization of the hybrid metalens. (a) Scheme of the imaging setup. Imaging results of (b1–b5) plano-convex lens, and (c1–c5) hybrid metalens. Tests of (b1) and (c1): Aluminum logo “HUST”, (b2) and (c2): hot soldering iron, (b3) and (c3): human’s hand, (b4) and (c4): upper body of a human, (b5) and (c5): full body of a human, with object distances of 0.1 m, 0.15 m, 0.5 m, 1.5 m, and 5 m, respectively. Scale bar: 2 mm.

We further performed five groups of imaging tests for both the hybrid metalens and LA9410-E3, analyzing objects like the aluminum logo “HUST,” a 200 °C hot soldering iron, and various human forms. Objects are positioned at different distances within a 24° FOV. Further details are available in Supplementary 9. Compared with LA9410-E3 in (Figure 4(b1)–(b5)), the hybrid metalens (Figure 4(c1)–(c5)) exhibit sharper edges and reduced blur. Taking the logo images in Figure 4(b1) and (c1) as an example, Figure 4(b1) shows clear blur and distortion due to the severe spherical and off-axis aberrations of LA9410-E3. In contrast, the hybrid metalens captures a clear image, demonstrating its aberration suppression capabilities. Additionally, the hybrid metalens effectively image objects at distances ranging from 0.1 m to 5 m. All images in Figure 4 have undergone contrast enhancement processing, standard in infrared thermal imaging. Raw images are available in Supplementary Material Figure A18. These results verify the hybrid metalens’ ability to correct for most aberrations in plano-convex lens.

## 4 Conclusions

In summary, we have experimentally demonstrated an aberration-corrected hybrid metalens in the longwave infrared, featuring a large diameter of 12.2 mm, an F/# of 1.2, and an FOV of 24°. Two metasurfaces correct the aberrations of the bare refractive lens, while the plano-convex lens shares most of the optical power to the hybrid metalens. Various optical experiments, encompassing MTF, transmission, and relative focusing efficiency, are performed on the hybrid metalens, yielding an average transmittance of 48.7 % and MTF values over 0.2 at 20 Lp/mm across full FOV. The hybrid metalens demonstrated average relative focusing efficiencies of 42.1 % at 9.3 μm and 34.6 % at 10.6 μm, more than double that of the plano-convex lens. Besides, the metalens-based thermal imaging system produces clear images over distances ranging from 0.1 m to 5 m, outperforming the plano-convex lens.

## Supplementary Material

Supplementary Material Details
