# Supplementary material for: Aberration-corrected hybrid metalens for longwave infrared thermal imaging
Source: Nanophotonics. 2024 Jun 3;13(17):3059–66. doi: 10.1515/nanoph-2023-0918 (PMC11502071; doi:10.1515/nanoph-2023-0918)
Supplement: Supplementary file 1 — Supplementary Material Details [file j_nanoph-2023-0918_suppl_001.docx]

Supplementary information for

Aberration-corrected hybrid metalens for longwave infrared thermal imaging

Tie Hu, ^1,†^ Liqinng Wen, ^1,†^ Haowei Li, ^1,†^ Shengqi Wang, ^2^ Rui Xia, ^1^ Zihan Mei, ^1^ Zhenyu Yang,^1^ AND Ming Zhao ^1*^

^1^Nanophotonics Laboratory, School of Optical and Electronic Information, Huazhong University of Science and Technology, Wuhan 430074, China

^2^ School of Engineering and Applied Science, Yale University, New Haven, Connecticut 06520, USA.

*Corresponding author: zhaoming@hust.edu.cn

1. The optimization of the hybrid metalens


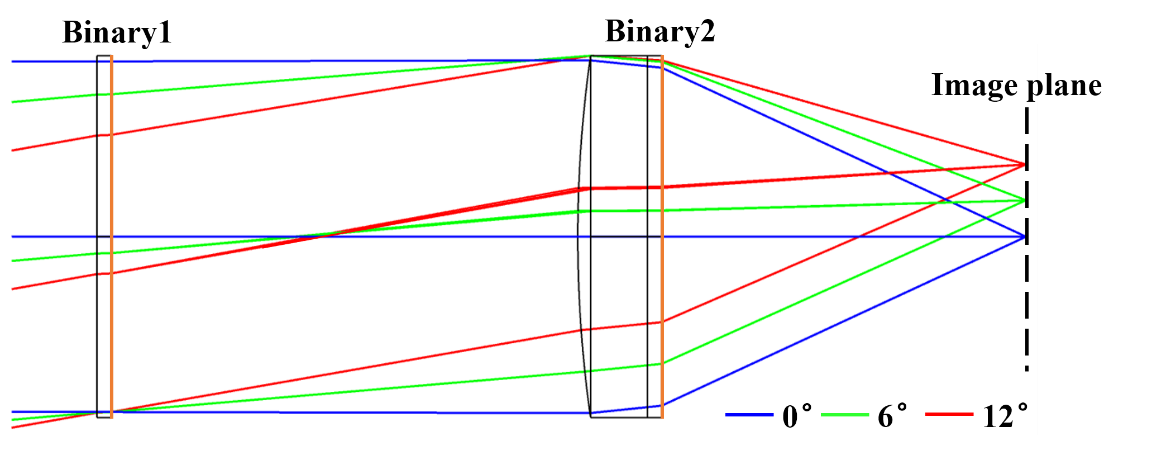


**Figure A1.** The model of the aberration-corrected hybrid metalens

**Figure A1** displays the optimization model of the aberration-corrected hybrid metalens in the 8-12 μm waveband, which is composed of two metasurfaces M1 and M2 denoted by orange lines and a commercial plano-convex lens (Thorlabs, LA9410-E3). Inspired by the Schmidt correction plate, M1 is initially positioned at the front focal plane of the plano-convex lens to correct off-axis aberrations. Also, M2 is placed on the flat rear surface of the plano-convex lens to compensate for residual aberrations. Optimization of the ideal phase profiles for the two metasurfaces was achieved using ray tracing techniques. Specifically, the binary 2 surfaces were selected to represent the metasurface in the commercial optical design software (Zemax OpticStudio), and the phase profiles are defined as even order polynomials of normalized radial coordinates:

$$\begin{aligned} \phi_{i}\left( \rho\right)=\sum_{j=1}^{3} a_{ij}\left( \lambda\right)\rho^{2j}\#\left( 1 \right) \end{aligned}$$

Where $\rho$ is the normalized radius of the metasurface, and $a_{ij}\left( \lambda\right)$ is the coefficient of the even-degree polynomial with a central wavelength of $\lambda$ , ***i*** is the metasurface serial number, and ***j*** is associated with even-order numbers. In this paper, the hybrid metalens adopts discrete multi-wavelength achromatic design, working at the wavelengths (8~ 12μm, in step of 0.5 μm). The coefficients $a_{1j}\left( \lambda\right)$ and $a_{2j}\left( \lambda\right)$ of metasurfaces M1 and M2 are optimized by minimizing the weighted root mean square (RMS) spot radii under different incident angles (0°, 5°and 12°). The optimized coefficients are listed in **Table A1**.

**Table A1.** The phase coefficients of two metasurfaces

| Design  Wavelength | Metasurface1 | | | Metasurface2 | | |
| --- | --- | --- | --- | --- | --- | --- |
|  | a_11_ | a_12_ | a_13_ | a_21_ | a_22_ | a_23_ |
| 8μm | -75.58 | 42.46 | 0.69 | -38.67 | 9.07 | 0.22 |
| 8.5μm | -71.15 | 39.96 | 0.65 | -36.62 | 8.54 | 0.21 |
| 9μm | -67.22 | 37.73 | 0.61 | -34.77 | 8.06 | 0.19 |
| 9.5μm | -63.69 | 35.75 | 0.58 | -33.10 | 7.64 | 0.18 |
| 10μm | -60.52 | 33.96 | 0.55 | -31.57 | 7.26 | 0.18 |
| 10.5μm | -57.65 | 32.34 | 0.52 | -30.17 | 6.91 | 0.17 |
| 11μm | -55.04 | 30.87 | 0.50 | -28.89 | 6.60 | 0.16 |
| 11.5μm | -52.65 | 29.52 | 0.48 | -27.72 | 6.31 | 0.15 |
| 12μm | -50.46 | 28.29 | 0.46 | -26.63 | 6.05 | 0.15 |

**Figure A2** shows the standard spot diagrams of the hybrid metalens at the focal plane. All the RMS radii of the hybrid metalens are within the diffraction limits at various incident angles and working wavelengths, while the RMS radii of plano-convex lens are greater than Airy radius. These results demonstrate the diffraction-limited focusing of the proposed hybrid metalens.


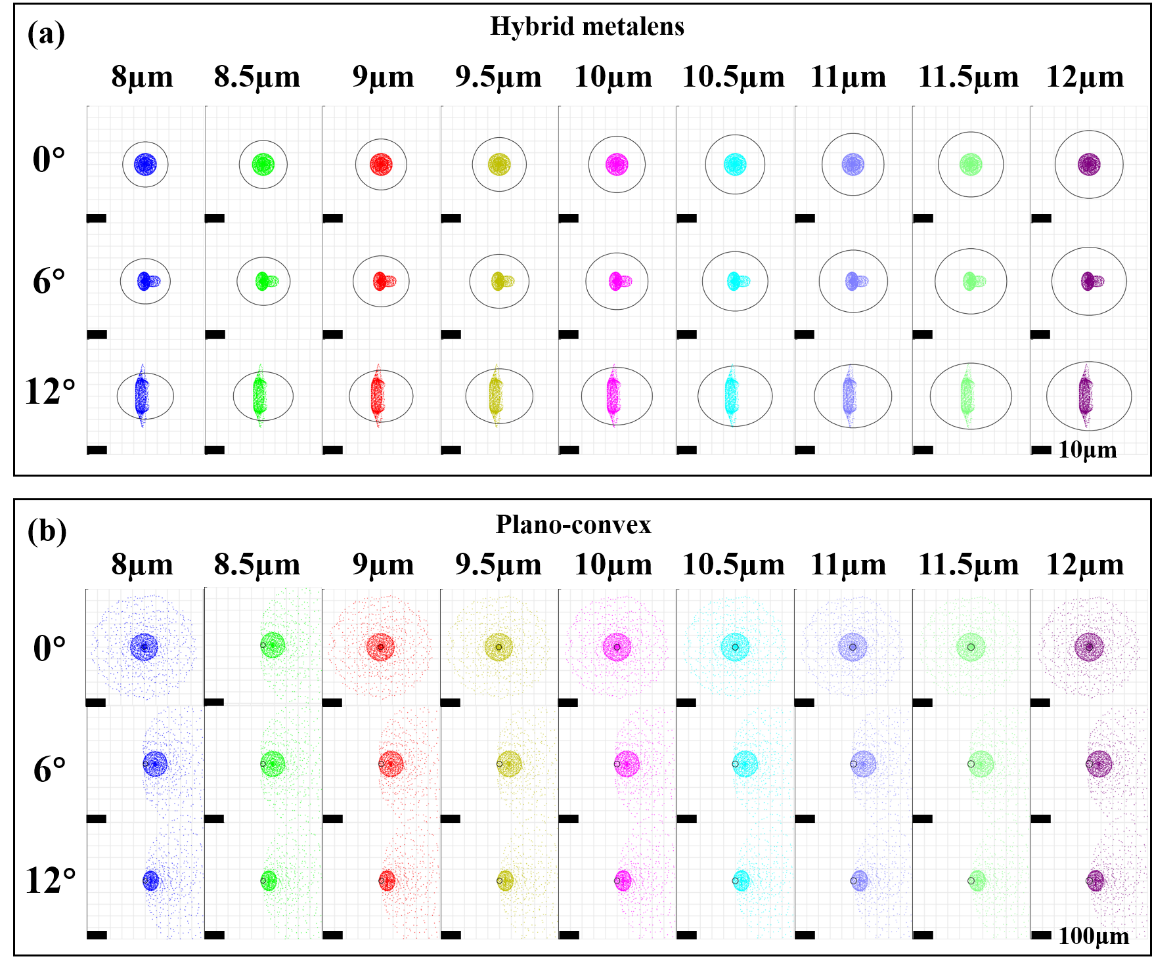


**Figure A2.** Standard spot diagrams for (a) hybrid metalens and (b) plano-convex lens at the focal plane. The rows of the figure represent the results at the incident angles of 0°, 6° and 12°. The columns of the figure represent the results of 9 different wavelengths. Scale bar:10 μm and 100 μm.

**Table A2** Comparison of RMS radius

| Wavelength(μm) | | 8 | 8.5 | 9 | 9.5 | 10 | 10.5 | 11 | 11.5 | 12 |
| --- | --- | --- | --- | --- | --- | --- | --- | --- | --- | --- |
| Airy Radius(μm) | | 11.6 | 12.3 | 13.1 | 13.8 | 14.5 | 15.3 | 16.0 | 16.7 | 17.4 |
| 0° | Plano-convex | 119.2 | 118.4 | 117.7 | 117.1 | 116.6 | 116.1 | 115.7 | 115.4 | 115.0 |
|  | Hybrid metalens | 3.8 | 3.8 | 3.8 | 3.8 | 3.8 | 3.8 | 3.8 | 3.8 | 3.8 |
| 6° | Plano-convex | 250.1 | 249.3 | 248.7 | 248.1 | 247.6 | 247.1 | 246.7 | 246.4 | 246.0 |
|  | Hybrid metalens | 3.2 | 3.2 | 3.2 | 3.2 | 3.2 | 3.2 | 3.2 | 3.2 | 3.2 |
| 12° | Plano-convex | 552.3 | 551.2 | 550.4 | 549.6 | 549.0 | 548.4 | 547.9 | 547.4 | 546.9 |
|  | Hybrid metalens | 10.4 | 10.5 | 10.5 | 10.5 | 10.5 | 10.5 | 10.5 | 10.5 | 10.5 |

To quantitatively analyze the efficiency of the hybrid metalens, we calculated its encircled energy and compared it with that of the commercial plano-convex lens (Thorlabs, LA9410-E3). The simulated results are listed in **Table A3**. While the broadband encircled energies of the plano-convex lens fall below 9% across the entire FOV, those of the hybrid metalens approach the diffraction limit. These results demonstrate the well aberration correction of the hybrid metalens. Here, broadband encircled energy represents the average of encircled energy across 9 discrete wavelengths.

**Table A3.** Comparison of the encircled energy

| Encircle radius$\left( \mu m \right)$ | | 6 | 8 | 10 | 12 |
| --- | --- | --- | --- | --- | --- |
| Diffraction limit | | 0.466 | 0.650 | 0.765 | 0.818 |
| Normal incidence | Hybrid metalens | 0.459 | 0.640 | 0.754 | 0.806 |
|  | Plano-convex | 0.049 | 0.068 | 0.081 | 0.087 |
| Oblique Incidence(12°) | Hybrid metalens | 0.231 | 0.356 | 0.47 | 0.565 |
|  | Plano-convex | 0.008 | 0.014 | 0.021 | 0.029 |

The Line Spread Function (LSF) data for broadband incident light in both the tangential and sagittal directions are obtained by computing an average of the optical intensity at 9 discrete wavelengths from 8 μm to 12 μm with a step of 0.5 μm. Then, the MTF, depicted in **Figures 1(b)** and **(e)** in the main text, is derived from the LSF using a one-dimensional Fourier transform. As for broadband geometric imaging simulation, 9 different configurations corresponding to 9 discrete wavelengths, were respectively established within the Zemax software’s Multiple Configuration Editor. Subsequently, derived from superimposing the simulated images of all configurations, are displayed in **Figures 1(c)** and **(f)**in the main text.

In summary, the hybrid metalens demonstrates significant aberration correction compared to the simulated results of the plano-convex lens.

1. Details of the meta-atoms design

As shown in **Figure A3**, five geometric types of meta-atoms—circle, cross, circle-inverse, ring, and cross-inverse—are used to construct the metasurfaces. The square nanopillar falls under the cross nanopillars category when widths w1 and w2 are equal. We sweep the geometric variables of the five kinds of meta-atoms, namely w1, w2, w3, r, r1, r2, and simulate their optical responses. Specifically, the radius (r) of circle nanopillar ranges from 0.25 μm to 1.25 μm. The widths w1 and w2 of cross nanopillar range from 0.5 μm to 2.5 μm. For circle-inverse nanopillars, w3 varies from 1.5 μm to 3 μm, and r from 0.6 μm to 1.25 μm. Ring nanopillars have an inner radius (r1) from 0.6 μm to 1.25 μm and an outer radius (r2) from 0.75 μm to 1.25 μm. For cross-inverse nanopillars, the outer width (w3) is 3 μm, with inner widths (w1 and w2) from 0.5 μm to 2.5 μm. All the critical dimensions of the designed meta-atoms exceed 0.5 μm. Periodic boundary conditions are applied in both X and Y directions, with perfectly matched layers in the Z direction. The simulation mesh order is set to 7. Anti-symmetric boundary conditions are applied along X min and X max directions, with symmetric conditions along Y min and Y max directions.


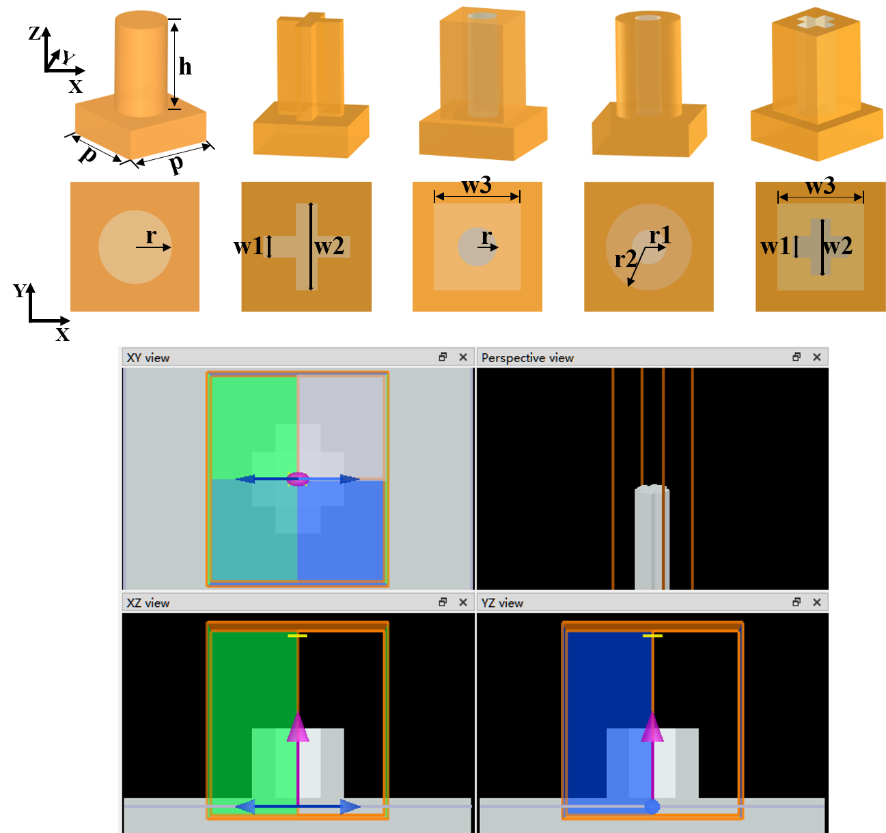


**Figure A3.** Details of meta-atom design.

1. The transmittance and phase profiles of two metasurfaces


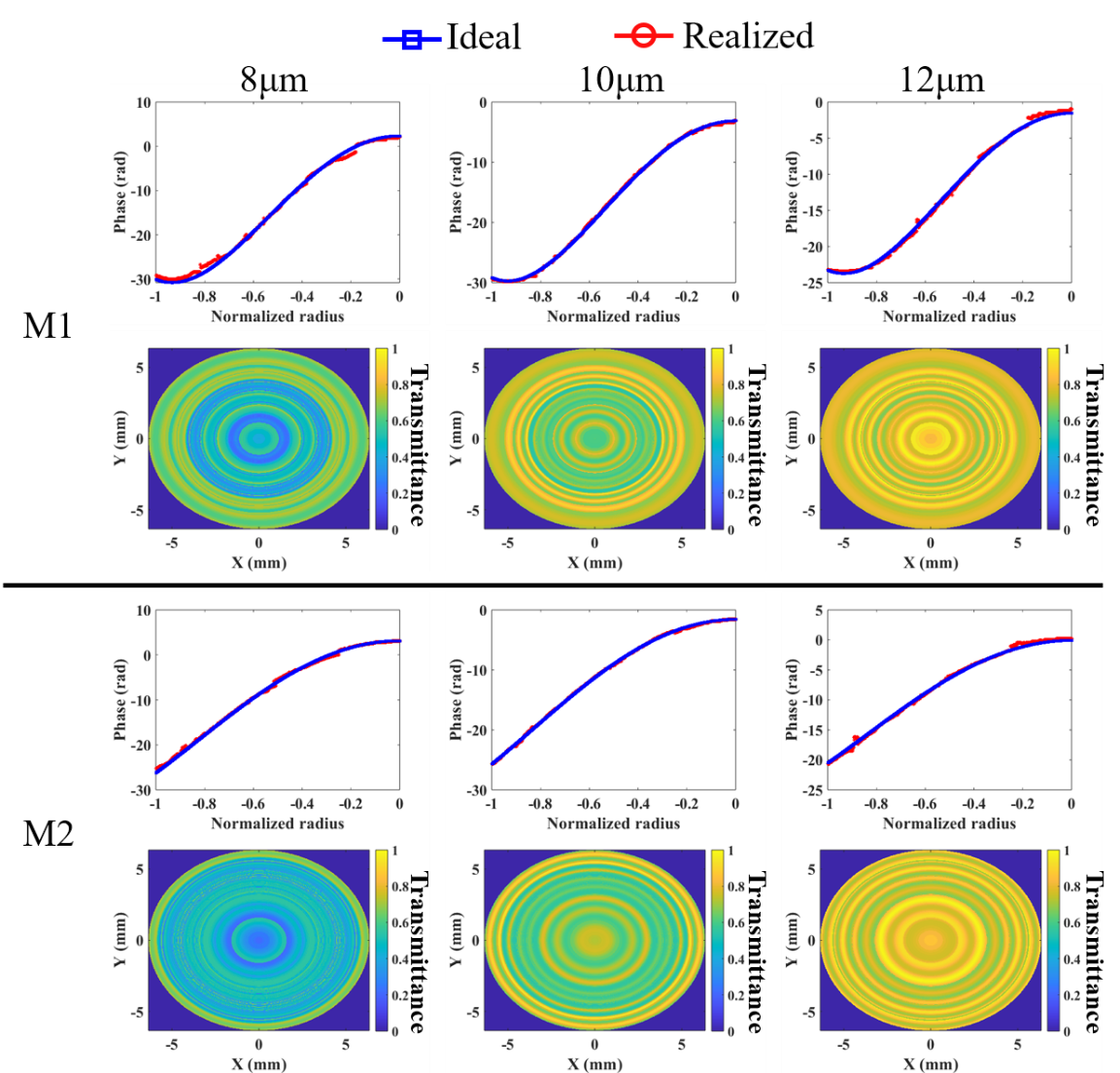


**Figure A4.** The transmittances and phase profiles of two metasurfaces in the hybrid metalens at the wavelengths of 8 μm, 10 μm, and 12 μm.

**Figure A4** depicts the realized phase and transmittance distributions. The average transmittances of metasurfaces M1 and M2 are respectively 71% and 68%. The lower transmittance at shorter wavelengths can be attributed to the diffraction loss of meta-atoms. Realized phases are consistent with the corresponding theoretical values, though there are slight deviations from theoretical ones due to the limited phase dispersion of the selected meta-atoms. **Table A4** shows the detailed root mean squares of the wave aberration function of both metasurfaces.

**Table A4.** RMS wave aberration function of two metasurfaces

| Wavelength (μm) | 8 | 8.5 | 9 | 9.5 | 10 | 10.5 | 11 | 11.5 | 12 |
| --- | --- | --- | --- | --- | --- | --- | --- | --- | --- |
| M1 | 0.057 | 0.022 | 0.012 | 0.014 | 0.011 | 0.01 | 0.009 | 0.009 | 0.013 |
| M2 | 0.032 | 0.016 | 0.011 | 0.008 | 0.009 | 0.008 | 0.01 | 0.012 | 0.017 |

1. Fabrication

The fabrication procedure involves two main processes: ultraviolet (UV) lithography and inductively coupled plasma (ICP) etching. Before fabrication, a 5" × 5" × 0.09" photomask blank (Telic Company) with a low-reflectivity chrome layer is used for reticle generation. Subsequently, a double-polished 2-inch germanium wafer undergoes meticulous preparation, including a thorough cleaning process. After preparation, an 800 nm thick layer of KMP C7600 i-line resist is meticulously deposited on the wafer using spin-coating, and it is subsequently subjected to a soft-baking process to achieve the requisite photoresist curing. The sample is then patterned using UV lithography with a Nikon-NSR-2205i11D stepper. It is then developed in tetramethylammonium hydroxide (TMAH) solvent. Subsequently, the exposed sample undergoes hard-baking. Afterward, the sample with its patterned photoresist layer is etched in an inductively coupled plasma system (Oxford Plasmalab, System100-ICP-180), using a proprietary high aspect ratio Bosch procedure. The process is concluded with the removal of the patterned residual photoresist through immersion in an N-Methyl pyrrolidone (NMP) solution, followed by stripping using oxygen plasma treatment facilitated by a Diener electronics PICO plasma stripper.


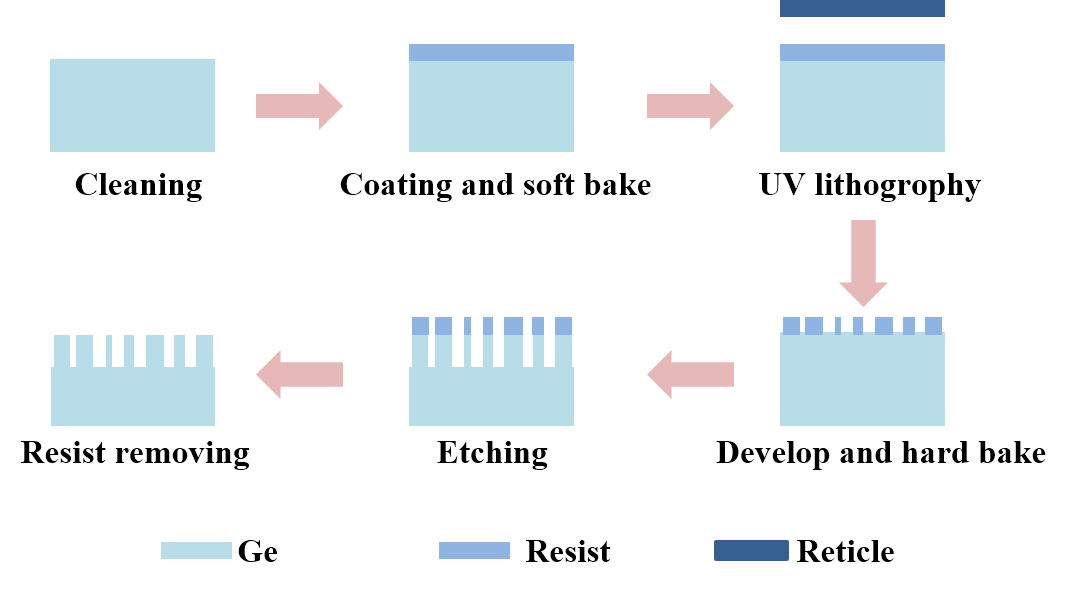


**Figure A5.** Fabrication procedure of the Ge metasurface.

1. Experimental feasibility of the hybrid metalens

We will demonstrate our approach practical by analyzing displacement tolerance and precision feasibility of our experimental mechanical devices. **Figure A6** illustrates our hybrid metalens model simulated with ray tracing. For simplicity, we move metasurface 1 (M1) along X and Z axes to analyze the transverse displacement ($\Delta_{X}$) tolerance and longitudinal displacement ($\Delta_{Z}$) tolerances individually. All simulations are conducted at a wavelength of 9.5 μm. The displacement range where value of encircled power at Airy radius (${Encir}_{Airy}$) decreases by half defines the tolerances.


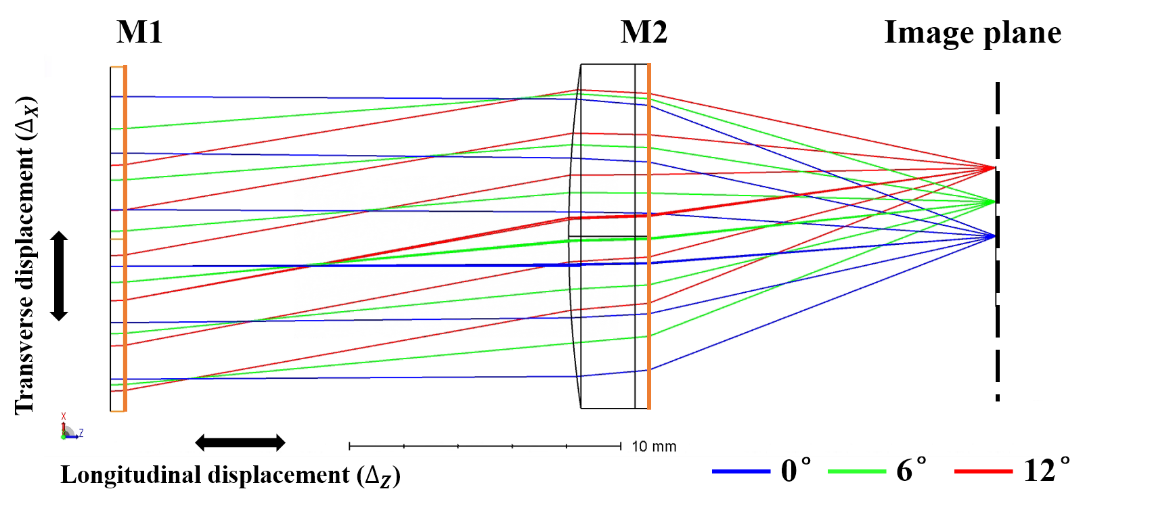


**Figure A6.** Simulation model of displacement tolerance

Encircled powers as functions of incident angle and transverse displacement ($\Delta_{X}$) are plotted in **Figure A7**. **Figures A7** (a), (b) and (c) illustrate how encircled power varies with encircled radius at three distinct incident angles. Generally, tencircled power decreases with increasing $\Delta_{X}$ and incident angle increase at each given radius. For ease of analysis, encircled powers at the Airy radius across varying $\Delta_{X}$ and incident angles are depicted in **Figure A7** (d). Across all three incident angles, all values of ${Encir}_{Airy}$ decrease to no less than half as $\Delta_{X}$ ranges from -0.3 to 0.3mm. Thus, the allowable transverse displacement tolerance is $\pm$0.3 mm.


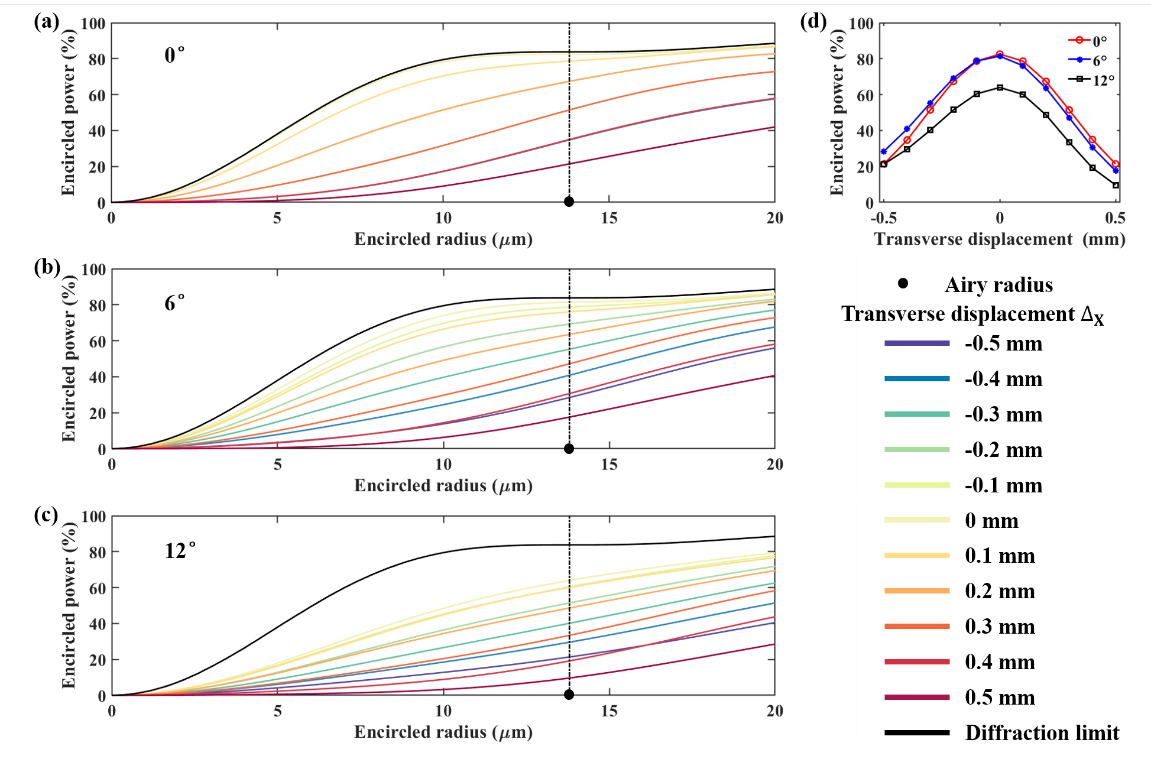


**Figure A7.** Analysis of transverse displacement tolerance. Encircled power as a function of encircled radius for incident angles of (a) 0°, (b) 6°, (c) 12°. Different colored solid lines represent transverse displacement $\Delta_{X}$ ranging from -0.5 to 0.5 mm in 0.1 mm increments. The black solid line denotes diffraction limit results. (d) ${Encir}_{Airy}$ with transverse displacement $\Delta_{X}$, marked by black dashed lines in (a), (b) and (c).

**Figure A8** illustrates the relationship between encircled powers, incident angle, and longitudinal displacement ($\Delta_{Z}$). **Figures A8** (a), (b) and (c) show encircled power variation with radius for three distinct incident angles. Typically, encircled power decreases as the absolute value of $\Delta_{Z}$ increases and with greater incident angles, at each specified radius. Encircled powers are notably stable under normal incidence. For analytical convenience, **Figure A8** (d) plots encircled powers at the Airy radius across varying $\Delta_{Z}$ and incident angles. For all incidences, all values of ${Encir}_{Airy}$ decrease by no more than half across $\Delta_{Z}$ from abouut -1.5 to 1.5 mm, indicating a longitudinal displacement tolerance of $\pm$1.5 mm. In summary, transverse and longitudinal displacement tolerances are $\pm$0.3 mm and $\pm$1.5 mm, respectively.


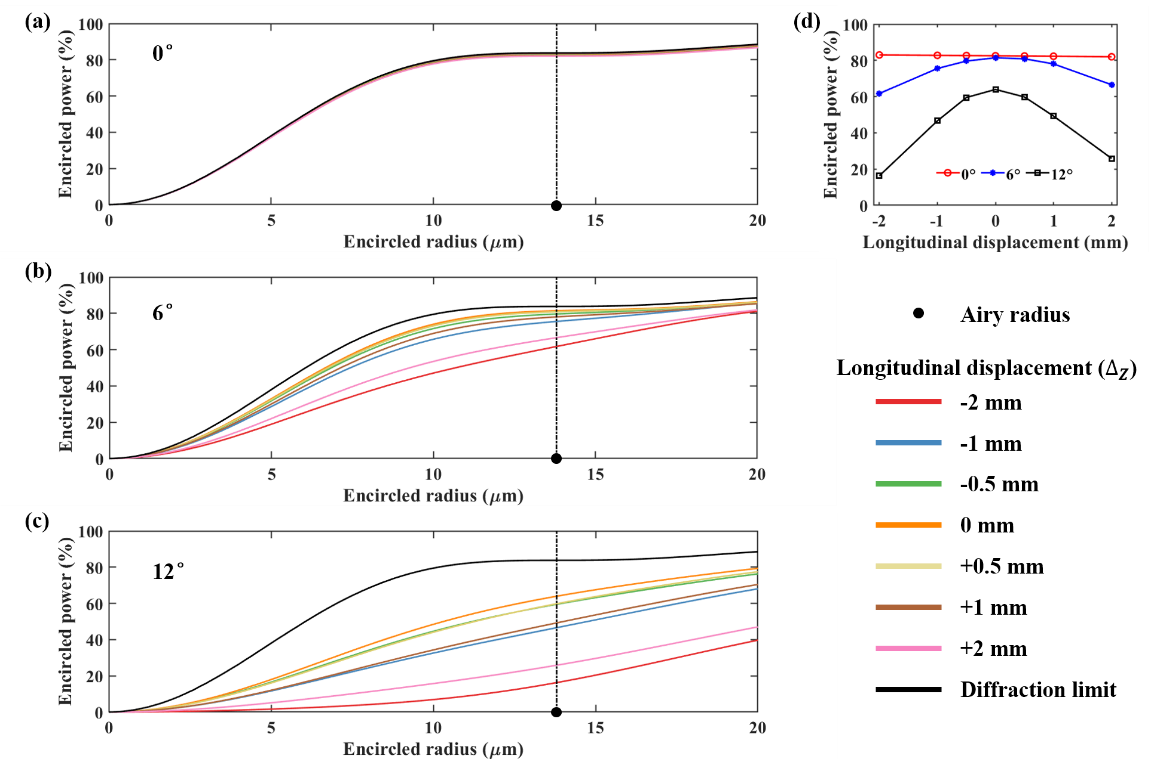


**Figure A8** Analysis of longitudinal displacement tolerance. Encircled power as a function of encircled radius for incident angles of (a) 0°, (b) 6°, (c) 12°. Different colored solid lines represent longitudinal displacement $\Delta_{Z}$, which rangs from -2 to 2 mm. Black solid line denotes the results of diffraction limit. (d) ${Encir}_{Airy}$ with longitudinal displacement $\Delta_{Z}$, marked by black dashed lines in (a), (b) and (c).

The feasibility of our concept will be validated by experimental mechanical fixture design. The mechanical fixtures of M1, M2 and plano-convex lens use CNC-fabricated SM05 threaded cage-mounted plate (CP32, Thorlabs). Specifically, to achieve the lateral alignment accuracy for the hybrid metalens, metasurfaces are mounted in grooves (see **Figures A9** (a) and (b)), matching the depth and horizontal dimensions of the fabricated metasurfaces. To prevent gaps between the refractive lens and M2, both are secured using with the SM05 threaded snap ring and a mechanical gasket. M1, refractive lens and M2 are assembled in a cage system. In summary, the mechanical components achieve a lateral alignment accuracy of about 0.2 mm, meeting the requirements for transverse displacement tolerance. To meet the hybrid metalens's longitudinal displacement tolerance, we first coarsely adjust the distance between M1 and the plano-convex lens with a micrometer, followed by fine tuning with a cage-mounted Z-axis displacement table (CT1A, Thorlabs), in increments of 1 µm. Conservatively, the achieved longitudinal displacement accuracy is within 1 mm. So, we strive to meet the mechanical accuracy requirements of assembly parts.


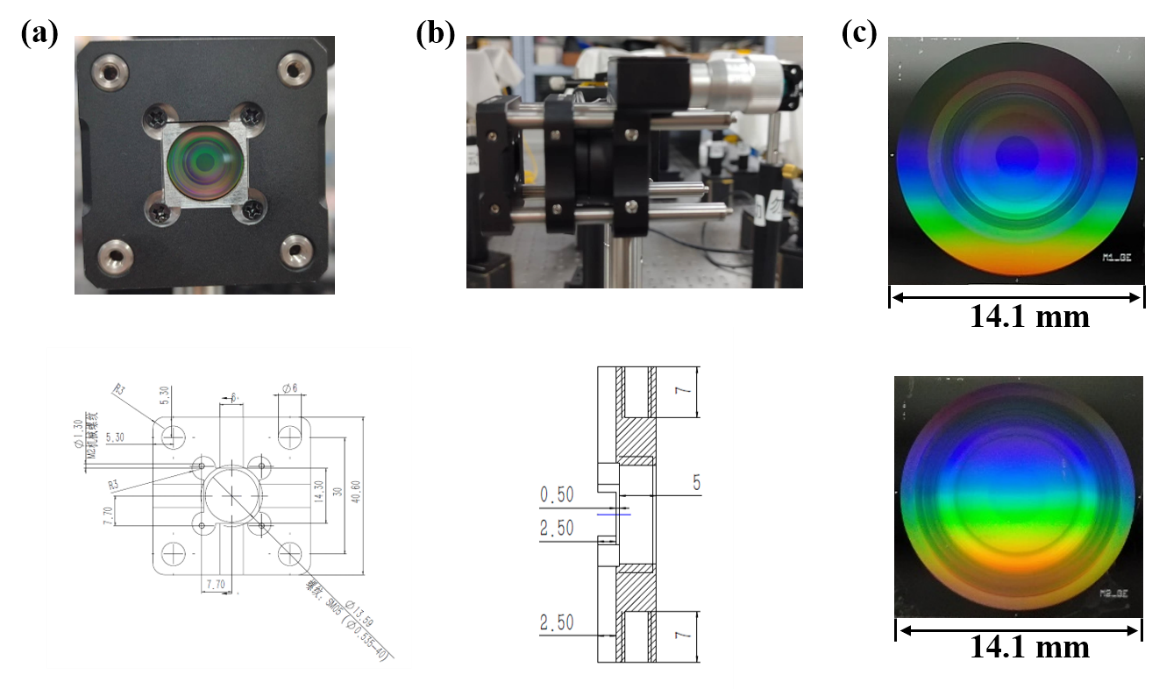


**Figure A9.** Design of the hybrid metalens mechanical fixture. (a) Mechanical fixture of M1, M2 and plano-convex lens. (b) Cage-mounted assembly of hybrid metalens. (c) Metasurfaces post-dicing.

1. The effects of fabrication errors and alignment errors

For simplicity, as seen in **Figure A6,** we will discuss the effect of alignment errors $\Delta_{X}$s on the MTF. All simulations are performed at a wavelength of 9.5 μm. MTF as functions of incident angle and transverse displacement ($\Delta_{X}$) are plotted in **Figure A10**. **Figures A10 (a), (b), (c)**, and **(d), (e), (f)** illustrate how MTF of sagittal and tangential ray vary with spatial frequency at three distinct incident angles. Generally, MTF decreases as the absolute value of $\Delta_{X}$ increases and as the incident angle increase at each given frequency. For ease of analysis, MTF of of sagittal and tangential ray at 20 Lp\mm across varying $\Delta_{X}$ and incident angles are depicted in **Figures A10 (g)** and **(h)**, respectively**.** Across all three incident angles, all $\mathrm{MTF}$ values decrease by more than half as $\Delta_{X}$ ranges from -0.5 to 0.5 mm. For example, as shown in **Figures A10 (g)**, MTF for 0° incidence decreases from 0.7 to 0.12 as $\Delta_{X}$ increases from 0 to 0.5 mm. These results verify that the alignment errors deteriorate the resolution of hybrid metalens.


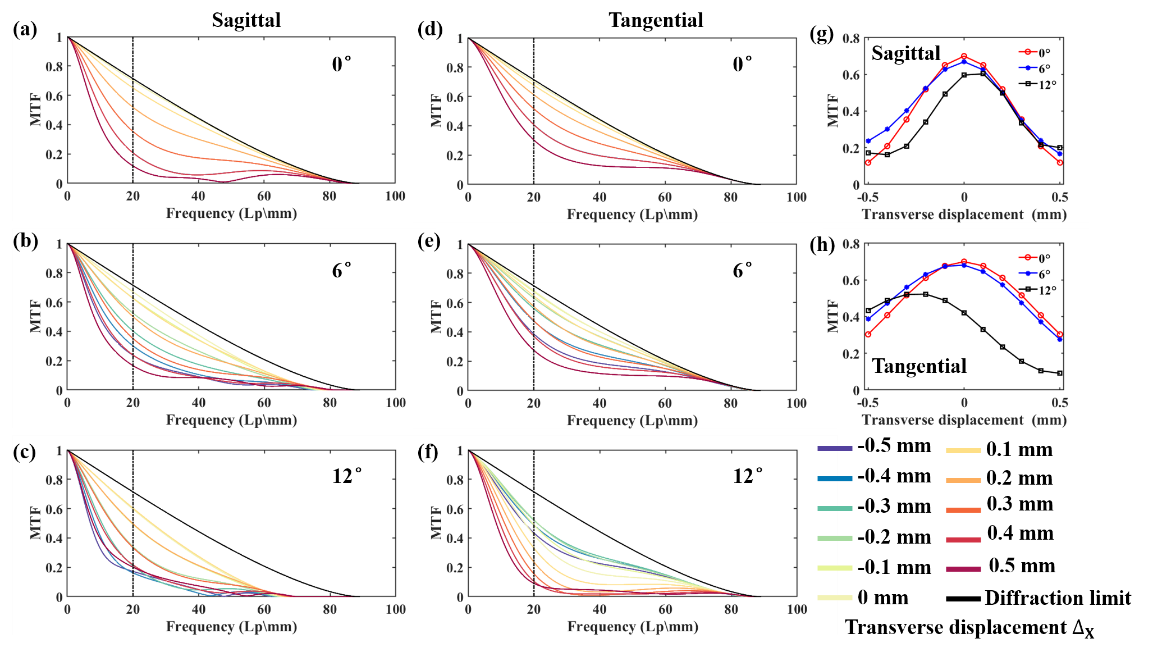


**Figure A10.** Effect of alignment error on the MTF of hybrid metalens. MTF of sagittal ray versus spatial frequency for the incident angles of (a) 0°, (b) 6°, (c) 12°. MTF of tangential ray as a function of spatial frequency for incident angles of of (d) 0°, (e) 6°, (f) 12°. Different colored solid lines represent transverse displacement $\Delta_{X}$, ranging from -0.5 to 0.5 mm in 0.1 mm increments. Black solid line denotes the results of diffraction limit. (g), (h) $\mathrm{MTF}$ at 20 Lp\mm versus transverse displacement ($\Delta_{X}$) marked by black dashed lines in (a), (b) and (c), (d), (e) and (f).

**Figure A11** shows the SEM iamges of various tested areas on the fabricated metasurfaces. The measured etching depths of the meta-atoms are detailed in **Figures A11** **(b)** and **(c)**. The measured etching depths vary: nanopillars from 18.3 to 15.5 μm with widths of 2.14 to 2.6 μm, and nanoholes from 13.26 to 16.25 μm with widths of 1.12 to 2.3 μm. This indicates a microload effect for each meta-atom type due to varying aspect ratios, as shown in **Figures A12** **(d)** and **(h)**). The microload effect leads to slight etching depth fluctuations in each geometric zone of both metasurfaces, with **Figures A12** **(c), (d), (g), (h)** showing consistent meta-atom types and minor aspect ratio variations. Generally, nanopillars have higher etching depths than nanoholes at similar widths, indicating intrinsic etching depth variances between the two meta-atom types. Considering the geometric types distributiions of meta-atom in both metasurfaces, **Figures A12** **(c)** and **(g)** suggest significant etching depth variations across each M1 and M2 zones. Additionally, etching depth variation of hybrid metalens exceeds 5 μm, potentially causing substantial phase fluctuations in both metasurfaces.


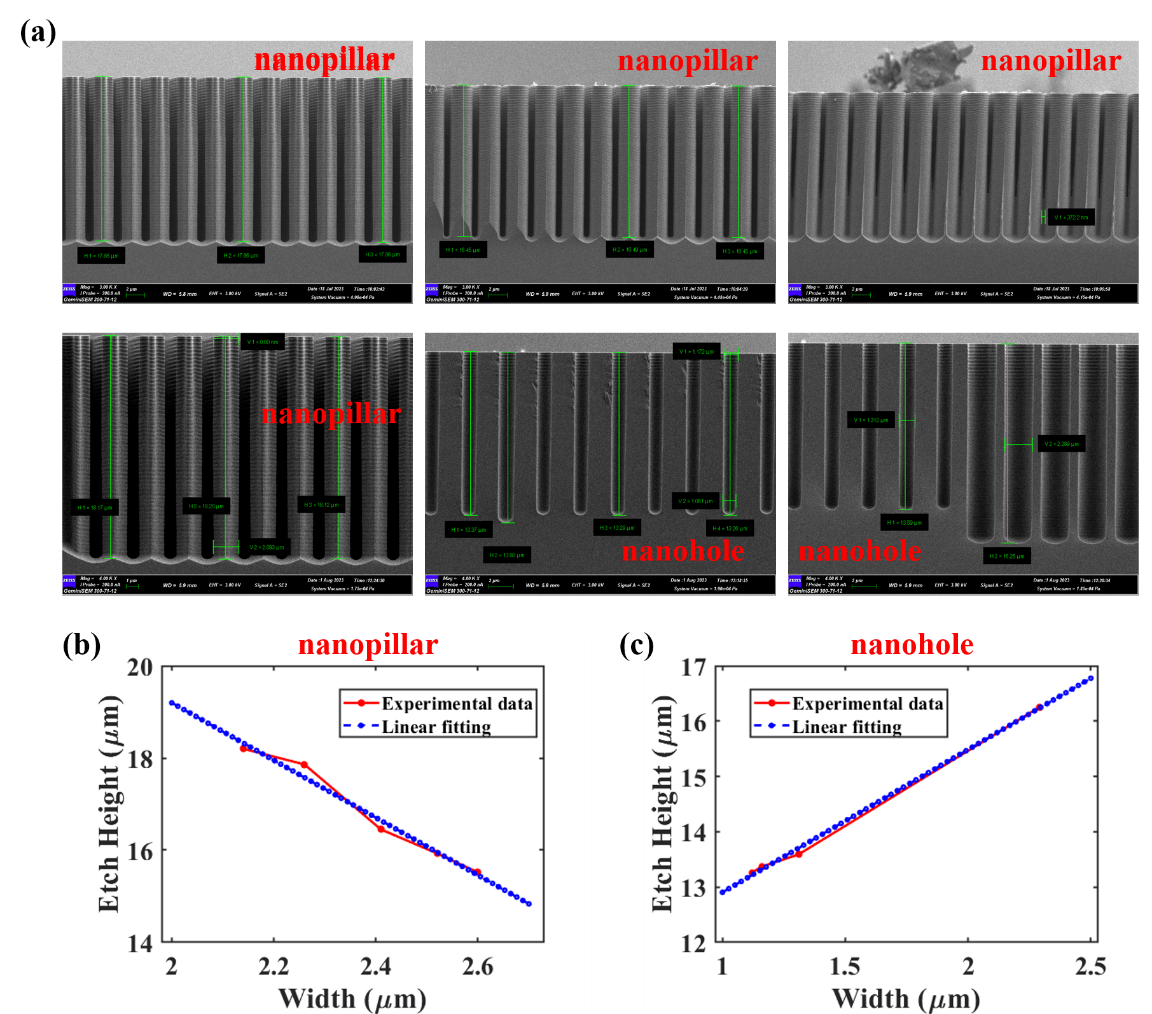


**Figure A11.** Fabrication errors of the hybrid metalens. (a) SEM of the fabricated metasurfaces. Mearured etching depths of (b) Nanopillar and (c) Nanoholes. The red solid and blue dashed lines denote the results of experimental datas and linear fitting, respectively.

The linear fitting is used to analyze the relationship between etching depth of meta-atoms and their width, with R square coefficients of 0.976 for nanopillar and 0.997 for nanohole. This indicates that the measured etching depths of nanopillars decrease linearly with their width, while those of nanoholes increase. As shown in **Figures A12** **(e)** and **(f)**, **(i)** and **(j)**, we can achieve the modified depth distribution of two metasurfacesm,which will be used to study the fabrication effects on the optical performances of hybrid metalens.


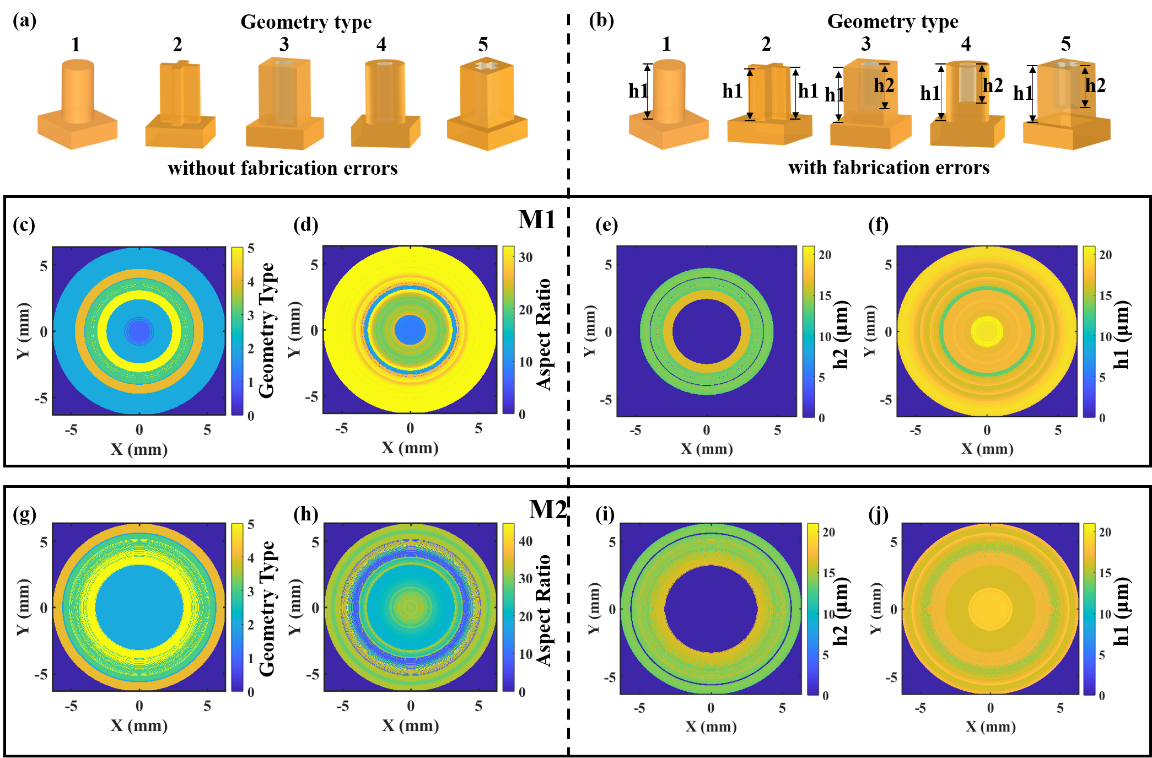


**Figure A12**. Geometric details of the hybrid metalens. Five geometric types of meta-atoms for (a) Without and (b) With fabrication errors. (c), (g) Geometric type profile of meta-atom and (d), (h) Aspect ration profile of M1 and M2, respectively. (e), (i) Inner height distribution and (f), (j) Outer height distributions of M1 and M2 with fabrication errors, respectively.

Given the mentioned fabrication errors, we simulate the optical responses of the hybrid metalens along a certain diameter direction using the FDTD method. **Figure A13** reveals significant fluctuations and deviations in the phase profiles of both metasurfaces with errors, compared to those error-free, across all wavelengths. These findings suggest that etching depth variations lead to notable phase fluctuations and errors, risking degraded optical performance. Regarding the impact on transmittance profiles, Transmittance profiles of M2 remains stable across all wavelengths. while M1 experiences a decrease at 8 μm and minor increases at 10 and 12 μm compared to its error-free counterpart.s.

**
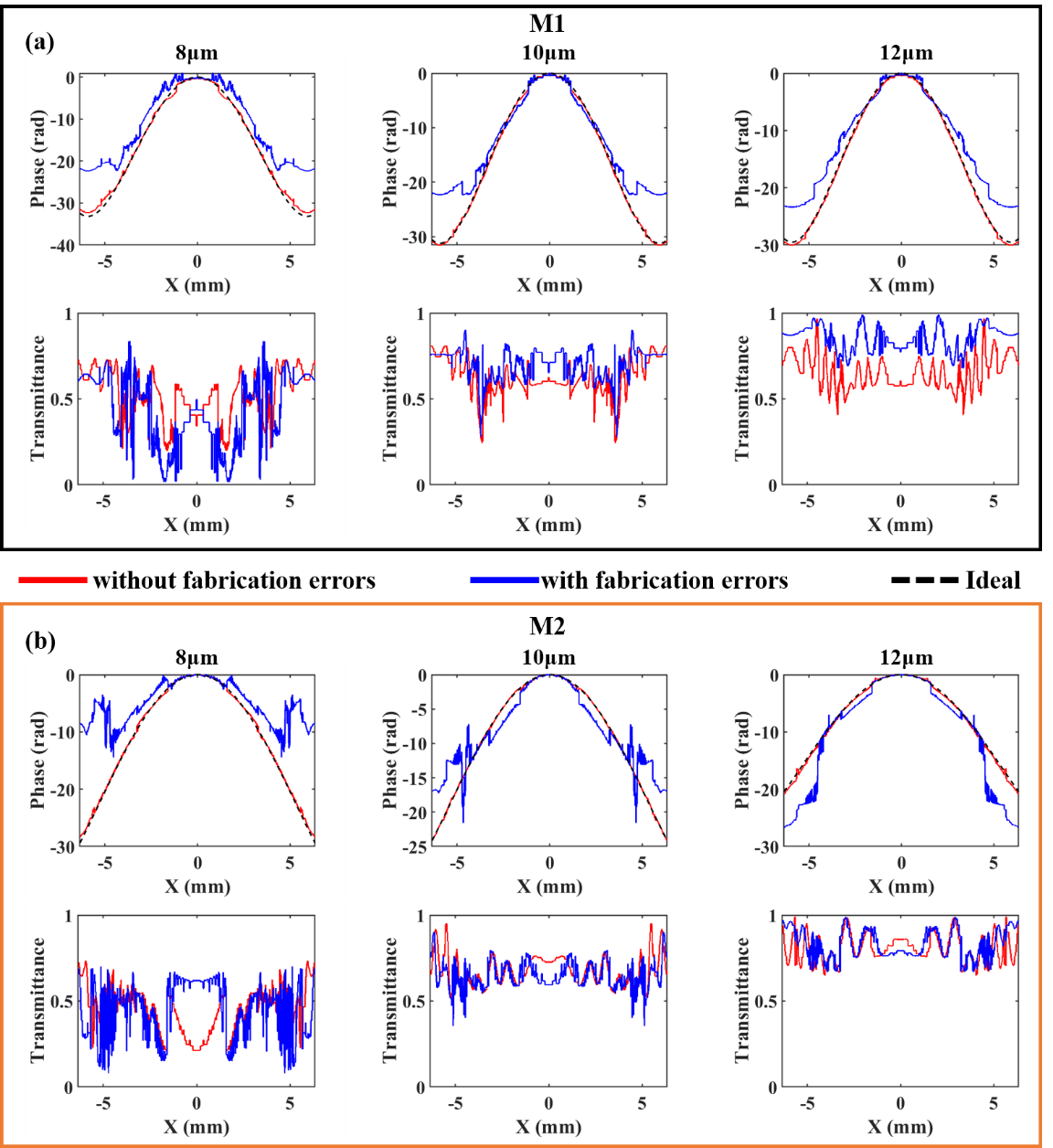
**

**Figure A13.** Effects of fabrication errors on optical response of hybrid metalens. Optical response comparisons of (a) M1 and (b) M2 in the hybrid metalens, respectively. Red and blue solid lines denote the results without and with fabrication errors, of theory, while black dashed lines denote theoretical results.

Afterwards, we examine the microloading effects on the optical performance of the unreduced hybrid metalens using a customized semi-vector simulation method. First, we fit the near-field phase of metasurfaces using even degree polynomials extracted from the FDTD-simulated meta-atom phase library. Subsequently, we import these coefficients into binary 2 surface to replicate the corresponding metasurface functionality. Finally, all optical performances of hybrid metalens is simulated by ray tracing. **Figure A14** **(b)**, **(e)** and **(h)** display broadband MTF curves for the hybrid metalens without the microloading effect, with the effect, and for LA9410-E3, respectively. The MTF of hybrid metalens with the microloading effect sharply drops to 0.1 at 10 Lp/mm, while without this effect, it gradually decreases to 0.1 at 52 Lp/mm across the full FOV, indicating a poorer resolution. Additionally, **Figure A14** **(c)** and **(f)** display alphabet letter chart tests over a 24° FOV, using broadband light. **Figure A14** **(f)** shows a severely blurred image from the hybrid metalens with microloading effect, contrasting with a sharp, minimally aberrated image from the lens without it in **Figure A14** **(c)**. Overall, the hybrid metalens with microloading effect has significantly inferior resolution and geometric imaging compared to the one without it. Notably, the resolution and geometric imaging quality of the hybrid metalens with microloading effect are comparable to those of the plano-convex lens, highlighting the ineffectiveness of aberration correction by the two metasurfaces.


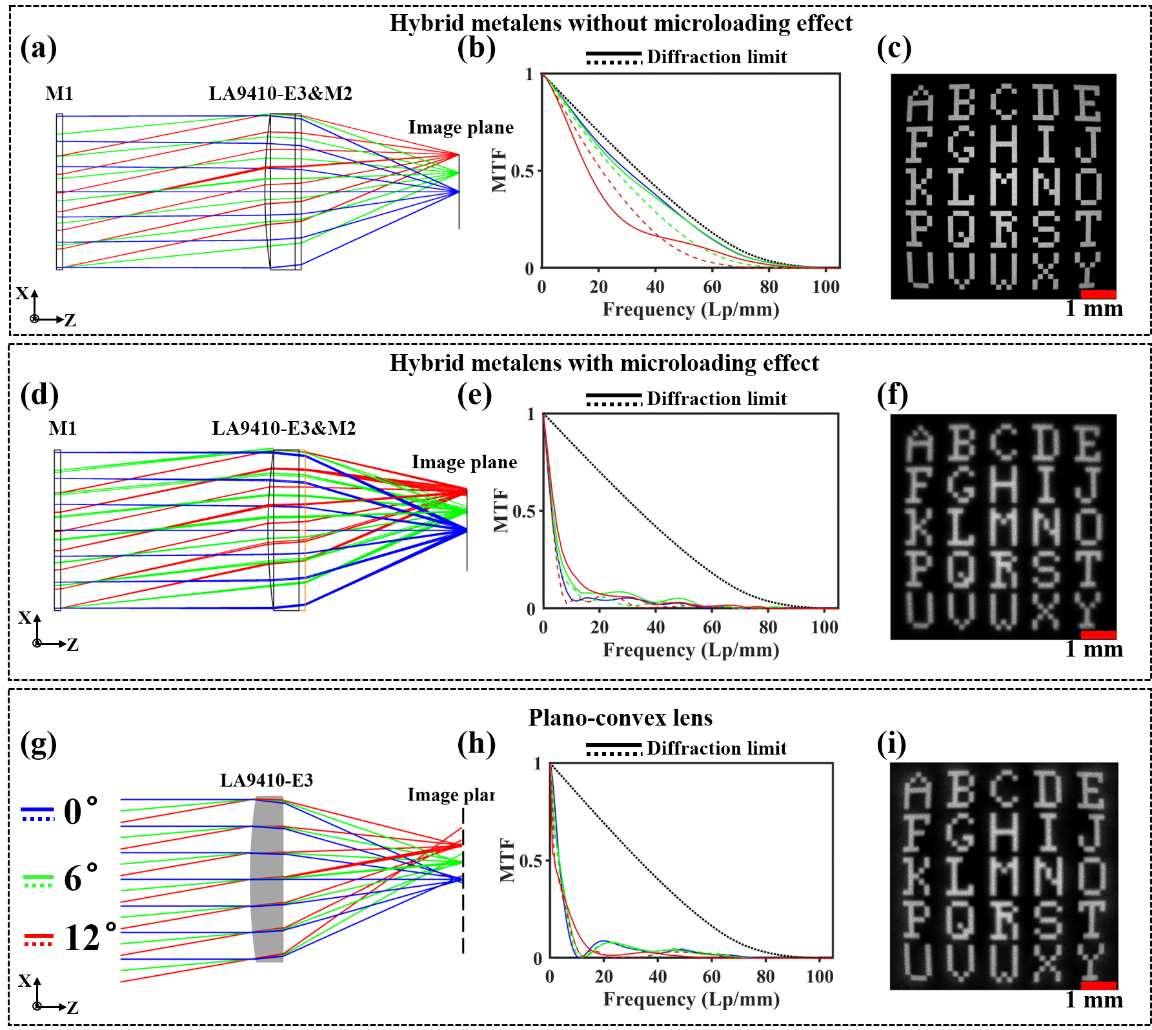


**Figure A14.** Microloading effects on hybrid metalens performances. 3D layouts: (a) hybrid metalens without microloading, (d) with microloading, (g) LA9410-E3. MTF curves vs. incidence angles for (b) without, (e) with microloading effect, (h) LA9410-E3. Blue, green, red, and black lines represent the results for incident angle 0°, 6°, 12°, and the diffraction limit, respectively Imaging characterizations: (c) without, (f) with microloading effect, (i) LA9410-E3.. Scale bar: 1 mm.

1. Focusing characteration experiment


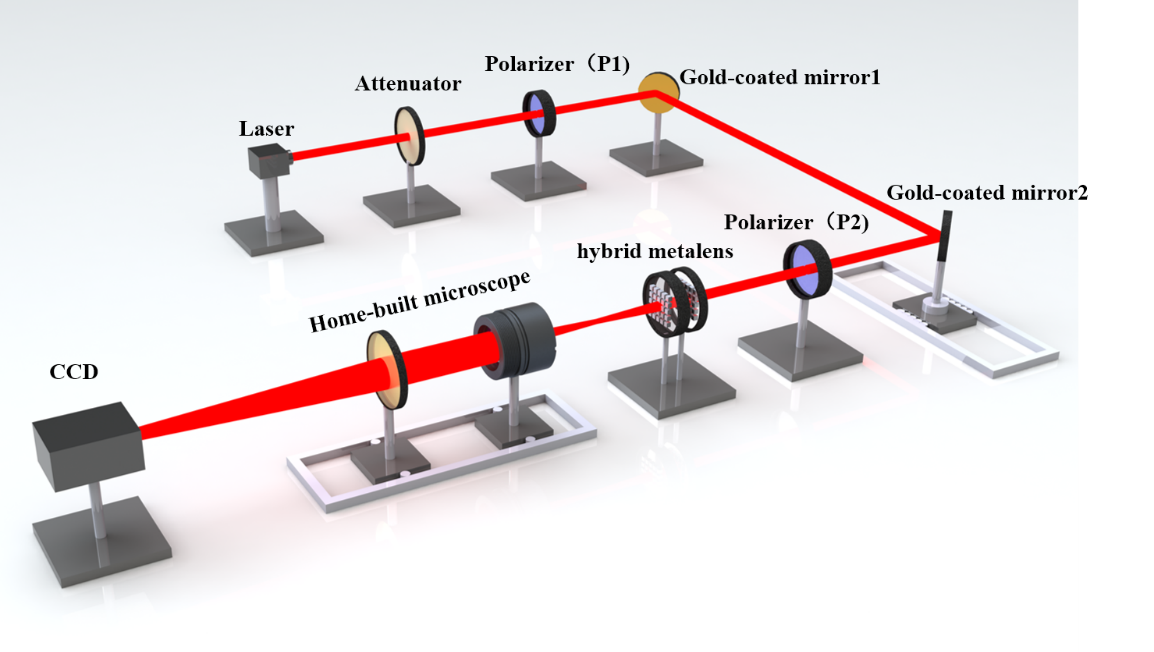


**Figure A15.** Scheme of the experimental set-up. The experimental system includes a $CO_{2}$ laser (center wavelength of 9.3 μm or 10.6 μm), a fixed attenuation ratio attenuator, two polarizers (P1, P2), two gold-coated mirrors, a home-built microscope, and a CCD.

In order to characterize the focusing properties of the hybrid metalens /plano-convex lens at different incident angles and wavelengths of the light source, the experimental set-up as depicted in **Figure A15** is established. This setup comprises an optical power attenuation system, a parallel beam deflection system, a home-built microscope, and an uncooled infrared focal plane detector (Wuhan Global Sensor, Plug617). The light source, a tunable $CO_{2}$ laser (Xiou laser, X30), has center wavelengths of 9.3 μm (50nm FWHM bandwidth) and 10.6 μm (200nm FWHM bandwidth). The optical power attenuation system consists of a fixed attenuation ratio attenuator (Thorlabs, NDIRW20 B) and a polarizer P1 (Thorlabs, WP25M-IRC). Two gold-doped mirrors (Thorlabs, PF10-03-M02) serve as integral components in establishing the beam deflection system. Notably, mirror M2 is under precise control through an electric linear displacement stage in conjunction with a manual rotary stage. The polarizer P2 is used to manipulate the polarization of incident light. The home-built microscope is made by an inverted thermal imaging lens (Thorlabs, TIL15) together with a plano-convex lens (Thorlabs, LA7028-E3).

Consider the experiment conducted at various incidence angles using a 9.3 µm wavelength as a representative case. Begin the experiment with incident light at 0°, calibrating the two mirrors to produce a collimated Gaussian beam that propagates perpendicular to the examined lens. To avoid camera saturation, calibrating the orientation of the two polarizers is essential. Subsequently, employ an electric displacement stage to continuously adjust the distance between the tested lens and the home-built microscope. The focal plane is identified as the position where the CCD detects the focused spot to be the roundest and brightest. After fixing the position of the lens being tested, intensity distributions at various incident angles can be captured by adjusting the beam deflection system's output angle. Moreover, upon tuning the laser to 10.6 μm, repeat these steps to gather focused spots at different incident angles.

1. Magnification calibration of the home-built microscopy

Samples with the designed logo are fabricated using the same process as shown in **Figure A5**, followed by the measurement of scanning electron micrographs. Upon substituting the metasurfaces with the target samples, the infrared detector is capable of capturing the magnified images. The magnification of the system, calculated by comparing the original and magnified lengths of the target, is determined to be 10.1x. Detailed experimental data are listed in **Table A5**.


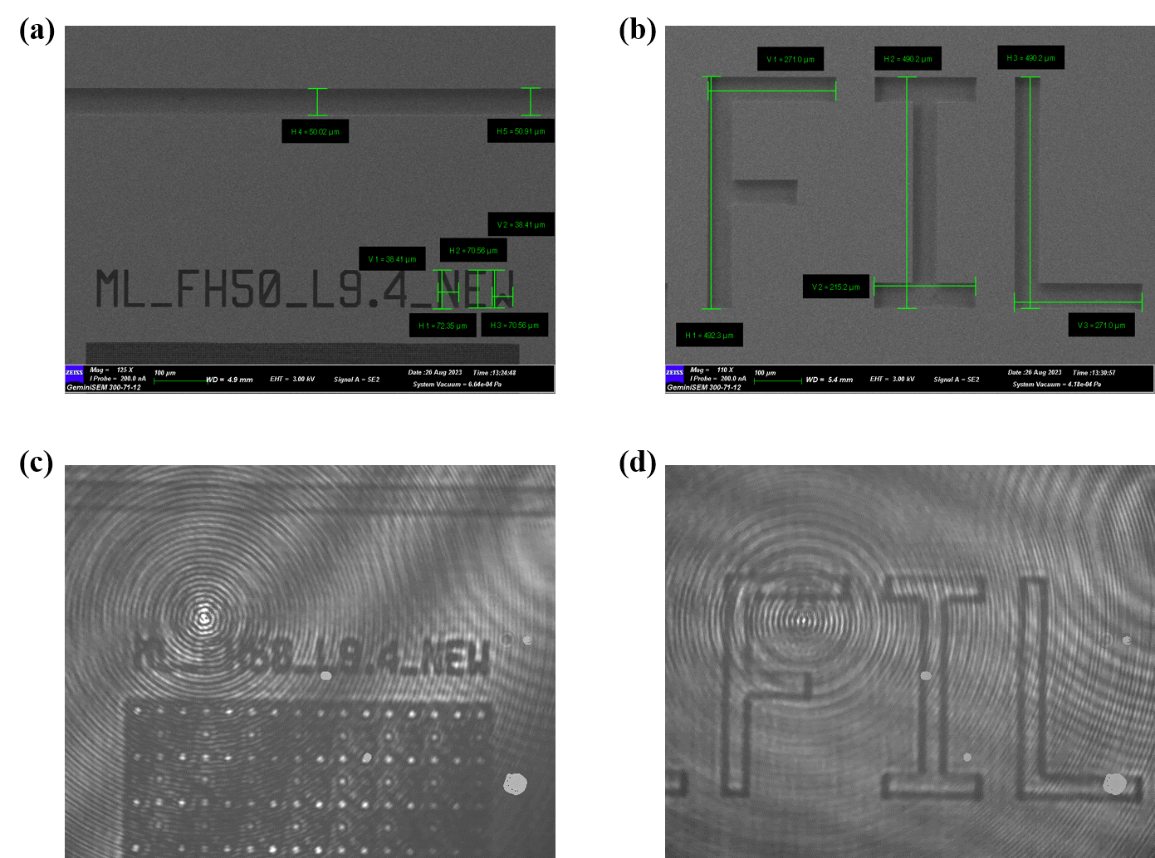


**Figure A16.** Magnification calibration of the home-built microscopy. (a),(b) Scanning electron micrographs (SEM) of the fabricated targets. (c),(d) Images captured by the infrared detector.

**Table A5.** Magnification calibration of the home-built microscope

| Experiment | Length (SEM) | Length (Magnified) | Magnification |
| --- | --- | --- | --- |
| 1 | 70.1 μm | 714.0 μm | 10.2x |
| 2 | 492.3 μm | 4947.0 μm | 10.1x |
| 3 | 492.3 μm | 4964.0 μm | 10.1x |
| 4 | 271.0 μm | 2788.0 μm | 10.3x |
| 5 | 271.0 μm | 2771.0 μm | 10.2x |
| 6 | 490.2μm | 4947.0 μm | 10.1x |

1. Measured MTF at different incident angles


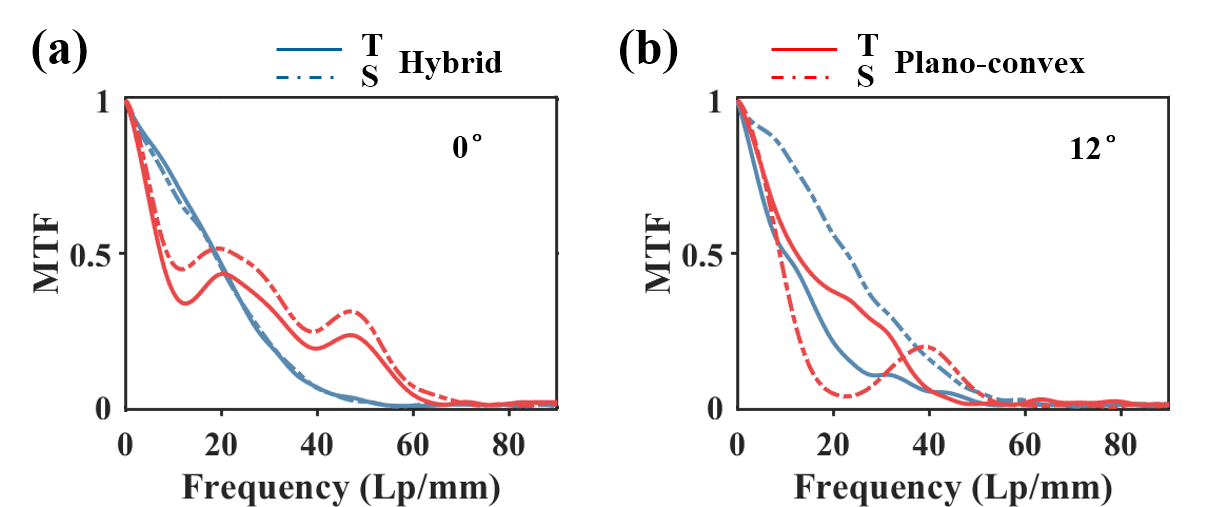


**Figure A17.** Polychromatic MTF of the two lenses at the incident angle (a) 0° and (b) 12°. Red and blue lines respectively represent the results of metalens and plano-convex lens. Solid and dashed lines are respectively the results in tangential(T) and sagittal(S) directions.

Polychromatic Line Spread Function (LSF) data in the tangential directions are obtained by averaging the optical intensity at wavelengths of 9.3 μm and 10.6 μm for each incident angle. Subsequently, the polychromatic MTF is derived by performing the one-dimensional Fourier transform of the polychromatic LSF, with results depicted in **Figures 4(b)** and **(c)** of the main text, as well as in **Figures A17(a)** and **(b)**. For both lenses, the MTF diminishes as the incident angle increases at each spatial frequency, consistent with the simulation results.

1. Achromatism characterization experiment

The chromatic aberration of the hybrid metalens is evaluated by assessing focal length shifts across various wavelengths. Initially, the position of the focal plane at a wavelength of 9.3 μm was determined by incrementally adjusting the distance between the tested lens and a custom-built microscope using an electric displacement stage. Subsequently, the focal plane position at a wavelength of 9.3μm was established as the reference plane (Z=0). The source wavelength is then adjusted to 10.6μm, and the electric displacement stage is employed to modify the Z plane, with intensity distributions recorded at varying Z planes. The $Z_{1}$ position of the focal plane at a wavelength of 10.6 μm corresponds to the stage displacement reading where the calculated Strehl ratio attains its maximum value. The focal length shift is determined by the difference from the reference plane (Z=0) established at a wavelength of 9.3μm. Furthermore, by replicating the aforementioned procedure, the focal length shift can be calculated using the focal plane position established at a 10.6μm wavelength as the Z=0 reference. Detailed data are recorded in **Table A6.**

**Table A6.** Experimental data of chromatism characterization


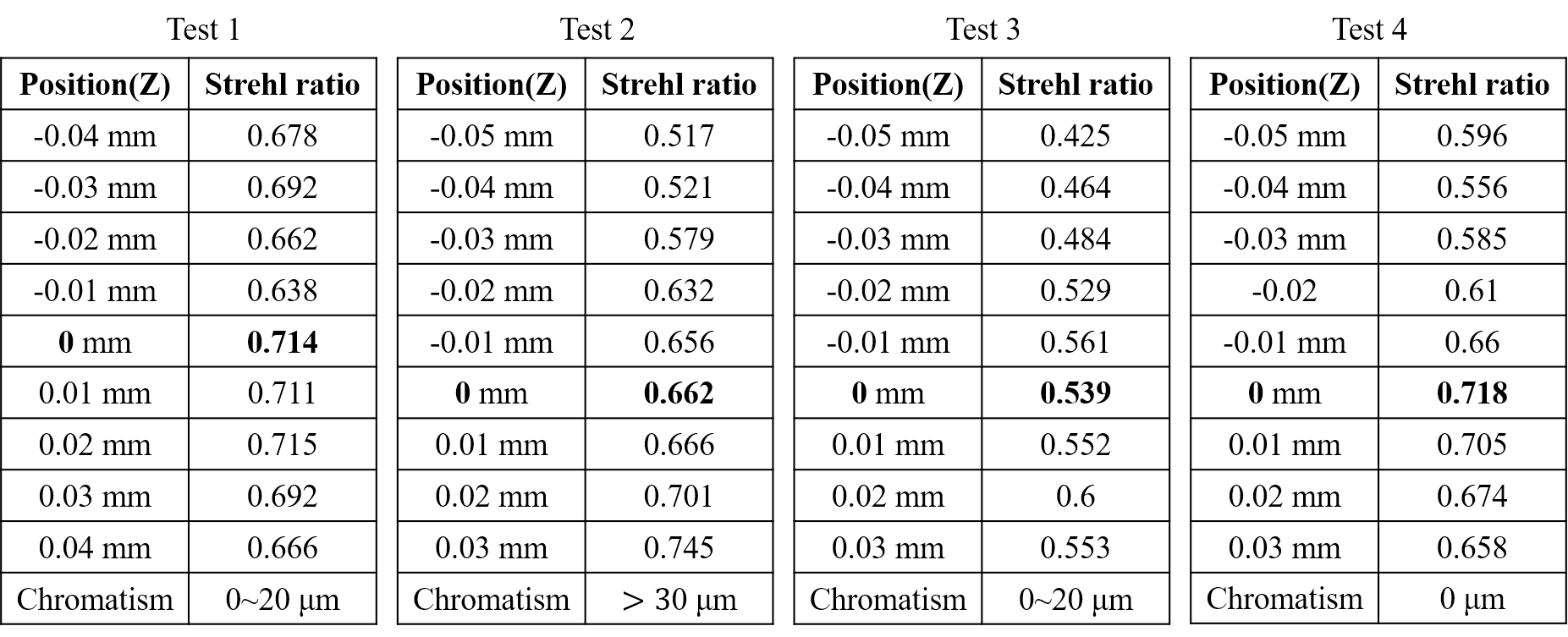


1. Imaging experiment

Five types of objects are selected: a hollow logo of HUST, heated by a blackbody and measuring 35mm in diameter (D2); a 200℃ electric soldering iron; a human hand; an upper body, and a whole human body. These objects are aligned with the optical axis of the tested lenses and detector. The distance between the test lens and the detector is adjusted using a 3D displacement stage. All experiments are conducted with a consistent FOV of 24°.

The object distance can be expressed as:

$object distance=\frac{D_{2}}{tan\frac{FOV}{2}}$ (6)

As depicted in **Figure A18**, the hybrid metalens is capable of imaging objects at varying distances. The hybrid metalens exhibits relatively low image contrasts, attributable to its lower transmittance (approximately 51.8%) in comparison to that of refractive lenses.


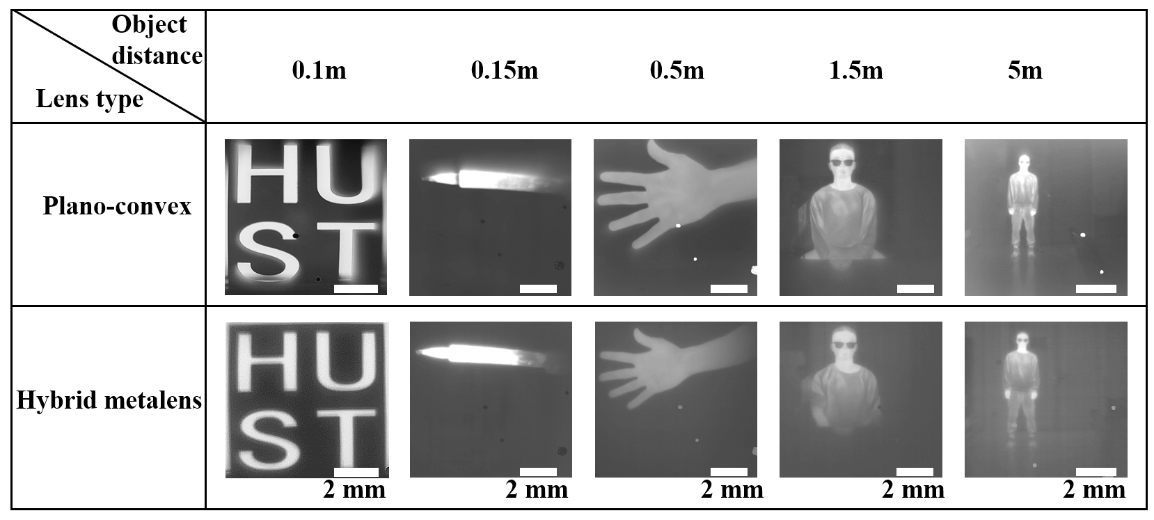


**Figure A18.** Row data captured by hybrid-metalens-based and plano-convex-lens-based imaging systems.
